# Improving lipid mapping in Genome Scale Metabolic Networks using ontologies

**DOI:** 10.1007/s11306-020-01663-5

**Published:** 2020-03-25

**Authors:** Nathalie Poupin, Florence Vinson, Arthur Moreau, Aurélie Batut, Maxime Chazalviel, Benoit Colsch, Laetitia Fouillen, Sarah Guez, Spiro Khoury, Jessica Dalloux-Chioccioli, Anthony Tournadre, Pauline Le Faouder, Corinne Pouyet, Pierre Van Delft, Fanny Viars, Justine Bertrand-Michel, Fabien Jourdan

**Affiliations:** 1 UMR1331, Toxalim (Research Centre in Food Toxicology), Université de Toulouse, INRAE, ENVT, INP-Purpan, UPS, 31300 Toulouse, France; 2 MetaToul-Lipidomic Core Facility, MetaboHUB, Inserm I2MC, 31000 Toulouse, France; 3 MedDay Pharmaceuticals, Paris, France; 4 Université Paris Saclay, CEA, INRAE, Médicaments Et Technologies Pour La santé (MTS), 91191 Gif-sur-Yvette, France; 5 Université de Bordeaux, CNRS, Laboratoire de Biogenèse Membranaire, UMR 5200, 33140 Villenave d’Ornon, France; 6 Université Clermont Auvergne, INRAE, UNH, Plateforme d’Exploration du Métabolisme, MetaboHUB Clermont, 63000 Clermont-Ferrand, France

**Keywords:** Metabolic networks, Ontology, Mapping, Lipidomics

## Abstract

**Introduction:**

To interpret metabolomic and lipidomic profiles, it is necessary to identify the metabolic reactions that connect the measured molecules. This can be achieved by putting them in the context of genome-scale metabolic network reconstructions. However, mapping experimentally measured molecules onto metabolic networks is challenging due to differences in identifiers and level of annotation between data and metabolic networks, especially for lipids.

**Objectives:**

To help linking lipids from lipidomics datasets with lipids in metabolic networks, we developed a new matching method based on the ChEBI ontology. The implementation is freely available as a python library and in MetExplore webserver.

**Methods:**

Our matching method is more flexible than an exact identifier-based correspondence since it allows establishing a link between molecules even if a different level of precision is provided in the dataset and in the metabolic network. For instance, it can associate a generic class of lipids present in the network with the molecular species detailed in the lipidomics dataset. This mapping is based on the computation of a distance between molecules in ChEBI ontology.

**Results:**

We applied our method to a chemical library (968 lipids) and an experimental dataset (32 modulated lipids) and showed that using ontology-based mapping improves and facilitates the link with genome scale metabolic networks. Beyond network mapping, the results provide ways for improvements in terms of network curation and lipidomics data annotation.

**Conclusion:**

This new method being generic, it can be applied to any metabolomics data and therefore improve our comprehension of metabolic modulations.

**Electronic supplementary material:**

The online version of this article (10.1007/s11306-020-01663-5) contains supplementary material, which is available to authorized users.

## Introduction

Small molecule profiling using complementary analytical setups now allows identification of hundreds of compounds in complex samples including both polar and nonpolar molecules. Metabolic profiling hence produces substantial lists of molecules that makes their biological interpretation long and tedious. One of the main challenges resides in connecting these compounds through metabolic reactions in order to associate metabolic fingerprints to modulations of metabolism (Frainay and Jourdan 2017). This contextual analysis can be achieved by embedding metabolic fingerprints in the context of Genome Scale Metabolic Networks (GSMN) since they aim at gathering all the metabolic reactions that can occur in a given organism or cell (Thiele and Palsson 2010). In particular, the Recon initiative is providing this GSMN for Human (Swainston et al. 2016; Thiele et al. 2013). Unfortunately, the task of mapping metabolites onto GSMN is still a challenge (Pham et al. 2019).

The crucial step in network-based analysis of metabolic fingerprints is to draw correspondences between identified molecules and nodes in the network. This question is far from being trivial since it requires harmonizing identifiers both in networks and datasets. Yet, metabolite names used in networks and in experimental datasets do not usually comply with any specific naming convention and come with different synonyms (e.g., α-*Ketoglutaric acid* and 2-*oxoglutaric acid*), spellings, formatting (lower or upper-case letters),… etc. Therefore, it is often unsuccessful to perform a direct and automatic matching between metabolite names or identifiers present in the network and metabolite names provided in datasets. Identifiers collected from metabolite databases such as KEGG (Kanehisa et al. 2014), ChEBI (Hastings et al. 2016), HMDB (Wishart et al. 2009, 2017) or Lipid Maps (Fahy et al. 2009) may be referenced in GSMN and therefore used to perform mapping between networks and datasets. Efficient methods and tools are available to convert identifiers [e.g., BrigeDB (van Iersel et al. 2010), Chemical Translation Service (CTS) (Wohlgemuth et al. 2010), UniChem (Chambers et al. 2013)] and thus allow adding identifiers in metabolic networks. For lipids however, this synonym-based matching often fails due to discrepancies between network and lipidomics datasets regarding the level of annotation provided for these compounds. In fact, both in lipidomics datasets and networks, the level of annotation ranges from fine grained definition (species) to general definition (classes) (Liebisch et al. 2013). For instance, when analyzing PC (diacylglycerophosphocholines) family, analytical tools will detect molecular species such as PC(34:1), PC(34:2), and in some cases PC(16:0/18:1) or PC(16:0/18:2), while in the human metabolic network Recon 2.2 (Swainston et al. 2016) all these molecules correspond to one single node (named “Phosphatidylcholine”). In contrast, chromatographic system usually does not allow the characterization of both C18w9:1 isomers while these isomers are present in the network (oleate or oleaniate) as two nodes. The challenge is thus to be able to automatically establish a correspondence between a lipid species and a lipid class during mapping.

The classification of lipids into classes can be described by an ontology. An ontology is a formal hierarchical representation of concepts, which can be used to describe elements from entities to abstract classes. Among the ontologies describing molecules some are focused on lipids like Lipid Maps while others are more generic like Chemical Entities of Biological Interest (ChEBI).

In this article, we propose a matching method using ChEBI ontology to bridge the gap between lipidomics data and GSMN. We will show how such ontology-based method can be used to better assess how the library built within the MetaboHUB consortium (French national Metabolomics and Fluxomics infrastructure) covers the human GSMN. Beyond this library mapping, the method had been applied to a lipidomics dataset produced on liver biopsies from healthy patients and from patients with non-alcoholic fatty liver (NAFL) and non-alcoholic steatohepatitis (NASH) (Chiappini et al. 2017). The implementation of this approach is freely available in a Python package and can be used online in MetExplore web server (Cottret et al. 2018).

## Material and methods

### Material

#### ChEBI ontology

ChEBI is a freely available repository of molecular entities focused on ‘small’ chemical compounds. The term ‘molecular entity’ refers to any constitutionally or isotopically distinct atom, molecule, ion, ion pair, radical, radical ion, complex, conformer, etc., identifiable as a separately distinguishable entity. ChEBI incorporates an ontological classification, whereby the relationships between molecular entities or classes of entities and their parents and/or children are specified. This is particularly well suited for the description of lipids. This publicly available database of chemical entities contains 56,090 annotated entities (release 179, September 2019).

#### Human metabolic network, Recon

The Recon2.2 metabolic network is one of the most curated human GSMN (Swainston et al. 2016). It is built on the annotated human genome and encompasses all of the metabolic reactions known to occur in any human cell or tissue. The Recon2.2 metabolic network includes 5324 total metabolites, corresponding to 2652 unique chemical compounds (or families) as some metabolites are duplicated to consider the compartmentation. A specific effort has been made in Recon2.2 to increase the annotation of metabolites with ChEBI identifiers, with 1256 of the unique compounds (47.4%) having associated ChEBI identifiers.

#### MetaboHUB Lipid Library

MetaboHUB gathers the complementary means (equipment, analytical techniques, software), expertise and competences of four French metabolomic facilities. Within MetaboHUB, lipidomics experts compiled a human lipidome database, which aims to reference a maximum number of identifiers per lipid analyzed in the consortium on plasma NIST (Bowden et al. 2017) (Supplementary Table 1). For each analyzed lipid we indicated the “Family”, “Class” and “Subclass” (as defined by Lipid Maps (LM) consortium), as well as a “Common Name” (usual name used for reporting), the molecular formula and different database identifiers (ChEBI, LM, PubChem (Kim et al. 2019), Swiss Lipids (SLM) (Aimo et al. 2015), InChI (Heller et al. 2015) and InChIKey). MetaboHUB database is built upon lipids analyzed by the consortium: fatty acids (saturated and unsaturated) are usually analyzed by gas chromatography coupled to flame ionization detector (GC–FID) or gas chromatography coupled to mass spectrometer (GC–MS), complex lipids such as glycerophospholipids, sphingolipids and bile acids are analyzed by liquid chromatography coupled to mass spectrometry (LC–MS), and Glycerolipids and Sterols can be analyzed with both techniques.

#### Liver lipidomics dataset

To highlight the benefit of the approach for lipidomics experimental datasets, we used a lipidomics dataset from the article by Chiappini et al. (2017). In this study, lipidomics analyses were performed on biopsies from normal liver, NAFL and NASH using classical liquid–liquid extraction and lipid profiling using, GC–MS and LC–MS. A random forest-based machine learning approach was used and allowed to identify a fingerprint of 32 lipids (Supplementary Table 2), discriminating NASH from healthy and NAFL livers. We manually retrieved the ChEBI identifiers for these 32 lipids and mapped them onto the metabolic network Recon2.2 in order to identify potential metabolic pathways and reactions related to NASH.

### Methods

#### Matching and mapping data

**Fig. 1:**
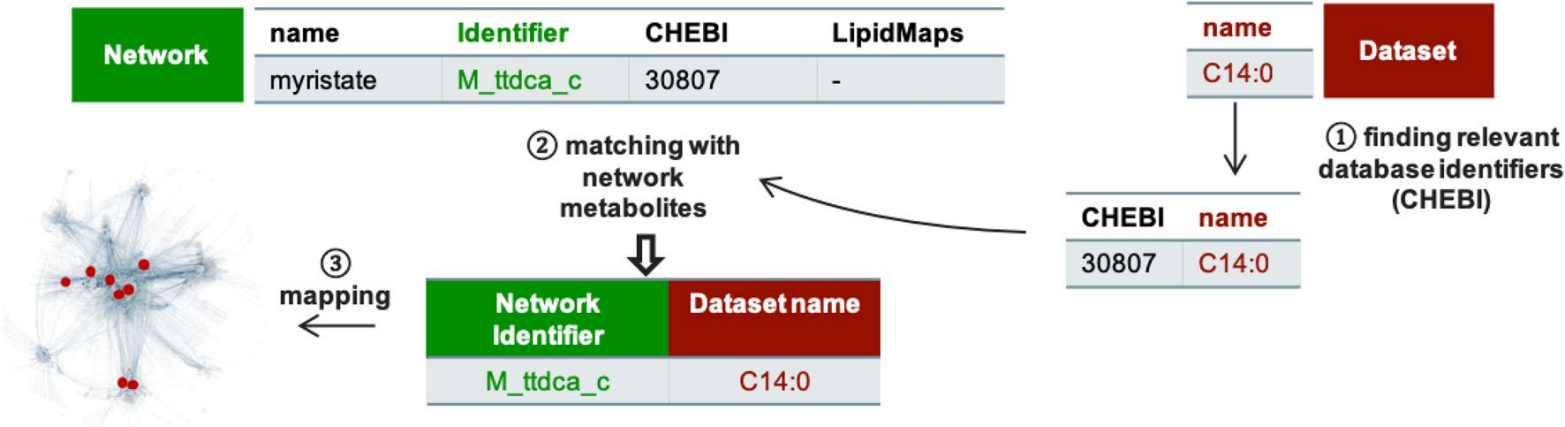
Steps required to perform metabolite/lipid mapping onto Genome Scale Metabolic Networks

The whole process required to analyze lipidomics data in the context of GSMN can be divided into two steps: (1) a matching step aiming at finding the metabolite identifiers in the metabolic network corresponding to each of the lipids in the dataset, (2) a mapping step consisting in positioning the lipid data onto the metabolic network (see Fig. 1). The matching step requires to find a correspondence between names or identifiers used to annotate lipidomics data and the ones informed in the network. The chemical names used on both sides are often different, preventing from doing a direct and automatic exact matching on names. Some public database identifiers can be informed for compounds in networks and datasets but, again, their type often differs. Hence, to perform matching it is necessary to use the same controlled vocabulary to annotate lipids in datasets and GSMN.

#### Finding relevant database identifiers

This step, Fig. 1, has to be done on both network and dataset molecules (or class of molecules). The aim is to select an identifier (e.g., ChEBI) which will be used to bridge the gap between the two lists of molecules. We chose to focus on the ChEBI identifiers since these are particularly well annotated in Recon2.2. These identifiers, like ChEBI, can be retrieved using services like CTS or BridgeDB.

#### Exact matching (name and ChEBI)

**Fig. 2:**
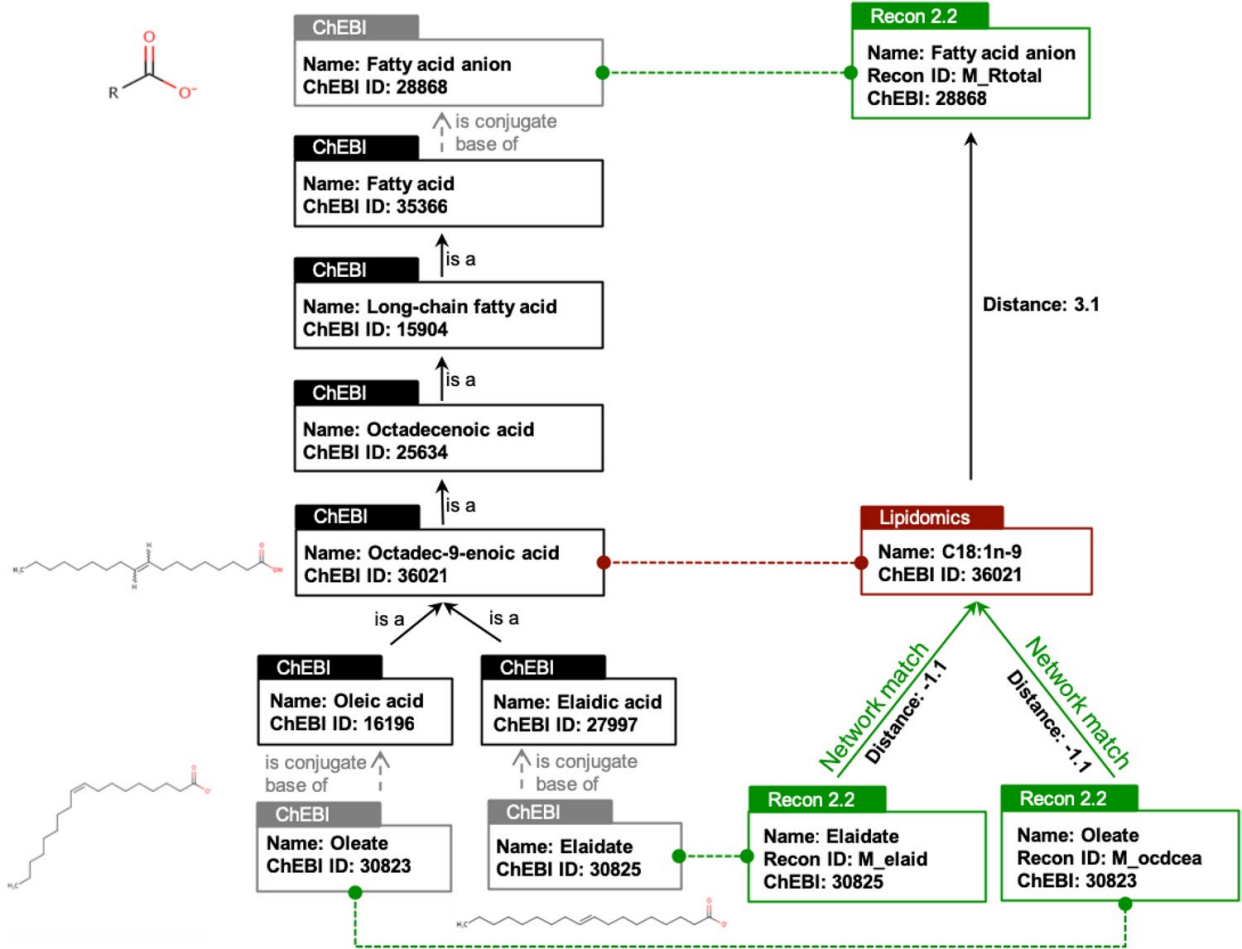
Example of lipid mapping. Relevant part of the ChEBI ontology is depicted on the left of the Figure (in black). Red box corresponds to the lipid annotated in Liver lipidomics dataset. Green boxes correspond to metabolites in Recon2.2 network

The matching step (Fig. 2) is called “exact” when, for a given lipid, there is the exact same identifier or name in the network and in the lipidomics dataset. In our application, exact matching was performed, first based on the names provided in the network and in the dataset, and then based on ChEBI identifiers.

#### Ontology based matching

As it will be shown in both examples, the exact matching is not sufficient to establish a correspondence for all molecules. Hence, we propose to complement the mapping pipeline by using an ontology-based matching (Fig. 2). This approach will allow to establish a link between molecules when the level of precision is different in GSMN and dataset (e.g., when only a class of lipids is present in the network, whereas the molecular species are detailed in the lipidomics dataset). For instance, regarding the glycerophospholipids, only the generic subclass phosphatidylcholine is present in the Recon2.2 network whereas more specific species (PC(32:0), PC(36:2), …) can be analytically measured and are therefore detailed in the dataset. Hence, we propose to link lipids in the network and in the dataset by using ChEBI ontology. Starting from the ChEBI identifier of a molecule in the dataset, the ChEBI ontology is scanned to see if downstream (more precise species) or upstream (more generic) ChEBI of metabolites present in the network can be found. To do so, the ontology is modelled by a graph (a directed acyclic graph more precisely) allowing to search paths between elements of the ontology and to measure lengths of these paths (number of edges between the source and target elements). Hence, we compute a distance representing, in the ontology, the distance between the metabolite in the dataset and the one finally retrieved in the network. An exact match is characterized by a matching distance of 0 between a lipid in the network and in the dataset. The distance is positive when the experimentally measured metabolite matches a more generic class in the network. On the contrary, the distance is negative when a more precise subclass of the observed metabolite is found in the network (see Fig. 2). The distance is slightly increased (by 0.1) when the anionic form (base) of the lipid is present in the network whereas the protonated form is present in the dataset (or vice and versa). Similarly, distances between tautomer molecules are increased by 0.01.

For instance, in Fig. 2, the “C18:1 n-9” fatty acid from the MetaboHUB dataset (octadec-9-enoic acid, ChEBI:36,021) matches with the network metabolite “M_ocdcea” (Oleate) with a distance of − 1.1 (“0.1” to account for the fact that oleate is a conjugate base of oleic acid, and “− 1” because oleic acid (octadec-9Z-enoic acid) is a specific octadec-9-enoic acid). The “C18:1 n-9” fatty acid also matches with the generic metabolite “M_Rtotal” (fatty acid anion) present in Recon2.2 with a distance of 3.1.

#### Matching method implementation

The matching method is implemented in a Python library (Metabolomics2Network) provided under CeCILL open source license. The library retrieves information from ChEBI ontology using libChEBIpy python library (Swainston et al. 2016a, b). The Directed Acyclic Graph structure of the ontology (made of ChEBI nodes and “is a” edges) is traversed to find shortest paths between lipids in the dataset and metabolites in the network. The library takes as input a json or text file with the lipids to be matched and a json file containing all the lipids from the network. The library returns, in a json or text file, the ontological closest metabolites in the network and the metabolites belonging to this shortest path in the ontology. If there are several nodes at equal distance, all of them will be retrieved (e.g., as shown on Fig. 2). The library is available at this URL: https://forgemia.inra.fr/metexplore/metabolomics2network.

In addition, the method can be accessed through MetExplore web server. Two outputs are available within MetExplore: an online grid showing the identifiers retrieved by the method with corresponding distances and a downloadable spreadsheet with more details on the matching. MetExplore is freely available and allows performing metabolomics data analysis in the context of GSMN. A tutorial is provided in Supplementary File 1.

#### Subnetwork extraction

**Fig. 3:**
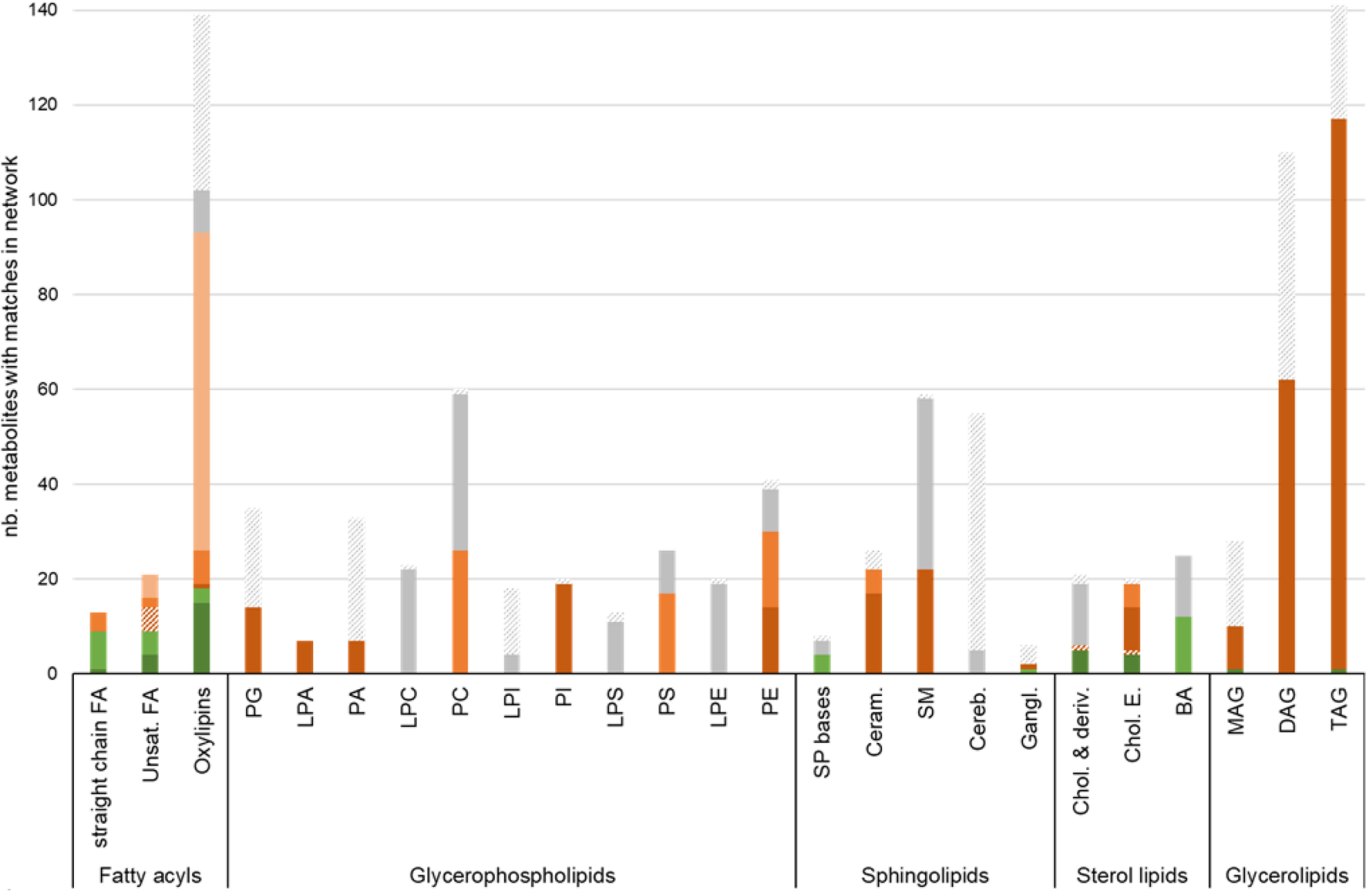
Ontology-based matching of MetaboHUB lipid library with Recon2.2 according to lipid classes. Bars represent the number of metabolites from the MetaboHUB library that have a match in Recon2.2. Exact matches and matches close to exact (with distance 0) are displayed in green and light green respectively. Matches on “parent” compounds with distance of 1, 2 or 3 and higher are displayed in different shades of orange, and matches with “child” compounds (distance of − 1) are displayed in hatched orange. Metabolites that have no matches in Recon2.2, although they have a ChEBI identifier, are displayed in grey while metabolites with no ChEBI are displayed in hatched grey

Once lipids are matched, it is possible to map them onto GSMN (Fig. 3). These lipids usually correspond to a few nodes among the thousand ones constituting the metabolic network (e.g., 16 nodes out 2652 for the liver lipidomics dataset) and the challenge is then to reduce the complexity of this large network to a more relevant sub-part (sub-network). Hence, we implemented in MetExplore a sub-network extraction method aiming at reducing the size of the network to ease the visual exploration of reactions connecting lipids in a lipidomics dataset. To extract a sub-network (subset of reactions) from the entire human metabolic network, we compute the union of lightest paths between each pair of mapped lipids. The lightest path between two nodes is the path with the lowest cumulated weight (defined as the sum of the squared degree of the nodes in the path (Croes et al. 2006)). This method is implemented in MetExplore web server (Cottret et al. 2018) and showcased in Supplementary File 1.

## Results

### Annotation of lipids in the MetaboHUB library

As lipids are complex molecules, we cannot get the same level of identification for all the studied classes. For instance, regarding unsaturated fatty acids and their metabolites, all the main molecular species can be analyzed and we were able to retrieve the most complete identifiers such as InChI, InChIKey, or even LM identifiers in the database. On the contrary, for complex lipids such as PC, the MetaboHUB consortium is currently not able to analyze all the single molecular species, especially to identify each specific fatty acid they contain (e.g., PC(16:0/18:1(11E)); PC(16:0/18:1(11Z)); PC(16:0/18:1(6E)); PC(16:0/18:1(6Z)); PC(16:0/18:1(9E)), and therefore only the different molecular entities of each subclass family (e.g., PC(34:1) with just the mention of the number of carbon atoms and unsaturations) have been referenced in the library. These molecular subclasses cannot currently be assigned to an InChI or InChIKey while ontology-based identifiers like ChEBI allow this abstraction. To summarize, for each entity, we adjusted the level of the identifier used for annotation to the accuracy of the analysis. The database compiles 968 analyzed lipid entities from 5 LM families and 21 LM subclasses. We retrieved a ChEBI identifier for 73% of them and a SLM for 61%, but only 32% have an InChI/InChIKey identifier, 28% a PubChem and 29% a LM (Table 1). Supplementary Table 1 contains the entire MetaboHUB database.

**Table 1:** Numerical compilation of the MTH Lipid Data SetLibrary

LM Family	MTH class	Number of entities	ChEBI ID	LIPID MAPS ID	PUBCHEM ID	SLM	InChIKey	InChI
Fatty acids	Straight chain fatty acids	13	13	13	13	12	13	13
	Unsaturated fatty acids	21	21	15	16	13	15	16
	Oxylipins	138	101	117	122	0	137	137
Glycerophospolipides	Glycerophosphoglycerols	35	14	0	0	35	0	0
	Lysophosphatidic acids	7	7	7	7	0	7	7
	Diacylglycerophosphates	33	7	0	0	33	0	0
	Lysophosphocholines	23	22	2	7	2	11	11
	Diacylglycerophosphocholines	60	59	16	0	57	0	0
	Lysophosphatidylinositols	18	4	18	18	18	18	18
	Diacylglycerophosphoinositols	20	19	0	0	0	1	0
	Lysophosphatidylserines	13	11	0	0	10	1	0
	Glycerophosphoserines	26	26	0	0	26	0	0
	Lysophosphatidylethanolamines	20	19	1	1	1	1	1
	Diacylglycerophosphoethanolamines	41	39	1	0	29	0	0
Sphingo lipides	Sphingoid bases	8	7	8	7	0	7	7
	Ceramides	26	22	21	21	26	22	22
	Sphingomyelins	59	58	1	0	18	13	13
	Cerebrosides	55	5	0	0	38	1	1
	Gangliosides	6	2	0	0	0	1	1
Sterol	Cholesterol and derivatives	21	19	18	11	2	20	20
	Cholesterol esters	20	19	19	19	0	19	19
	Bile acids	25	25	24	25	0	25	25
Glycerolipides	Monoacylglycerols	28	10	1	1	27	0	0
	Diacylglycerols	110	62	0	0	110	0	0
	Triacylglycerols	141	117	0	0	137	0	0
Total entities		968	709	283	146	594	312	311
*% from the total*			*73*	*29*	*15*	*61*	*32*	*32*

### Exact matching on Recon2.2 with names and with ChEBI identifiers

A first matching of the 968 molecules of MetaboHUB Lipid Library on Recon 2.2 using lipid names gave only 3 exact matches for 14,15-DiHETE, 9(10)-EPoMe and 5-HEPE, while exact matching using ChEBI produces 31 matches. The poor efficiency for name-based matching is mostly due to the fact that lipids are often called with different names: for instance, the well-known docosahexaenoic acid (DHA) has several synonyms (e.g., Cervonic acid or C22:6n-3,6,9,12,15,18 acid). Using ChEBI, we were able to match only 4.5% of the identified lipids from the Library to Recon2.2 network. These 31 exact matches concern oxylipins and few fatty acids which are simple molecules for which the studied molecular species are exactly the same in Recon2.2 and in the Library. For other molecules, this absence of matching is mainly due to the different level of characterization of lipids between network and lipidomic dataset.

### Ontology-based matching of the spectral library

By using the ChEBI ontology rather than the exact ChEBI identifier, we can match the lipid species from the MetaboHUB library with more generic lipid classes defined in Recon2.2. The ontology-based matching enables to match 492 more lipids from the MetaboHUB Library on Recon2.2, representing almost 70% of the lipids having ChEBI identifiers (51% of all the lipids) (see supplementary file 3 for matching results). The result of the matching differs largely depending on the lipid classes (see Fig. 3): almost all glycerolipids (98.9%) with ChEBI identifiers (67% of all glycerolipids) are matched on Recon2.2 with a distance of 1, whereas only about 53% of sphingolipids and glycerophospholipids (with ChEBI identifiers) are matched with a distance of 1 or higher. This heterogeneity in the matching results among lipid classes allows to highlight some classes or subclasses that are less well described or even missing in the metabolic network. Regarding glycerolipids, the 3 classes measured in MetaboHUB (namely, monoacylglycerols, diacylglycerols and triacylglycerols) are represented in Recon2.2 by only one generic metabolite for each class: monoglyceride (“mag_hs”), diglyceride (“dag_hs”) and triglyceride (“tag_hs”). In comparison, some sphingolipids and glycerophospholipids that are present in the MetaboHUB library are missing in Recon2.2, such as the cerebrosides and all the lysoglycerophospholipids.

**Fig. 4:**
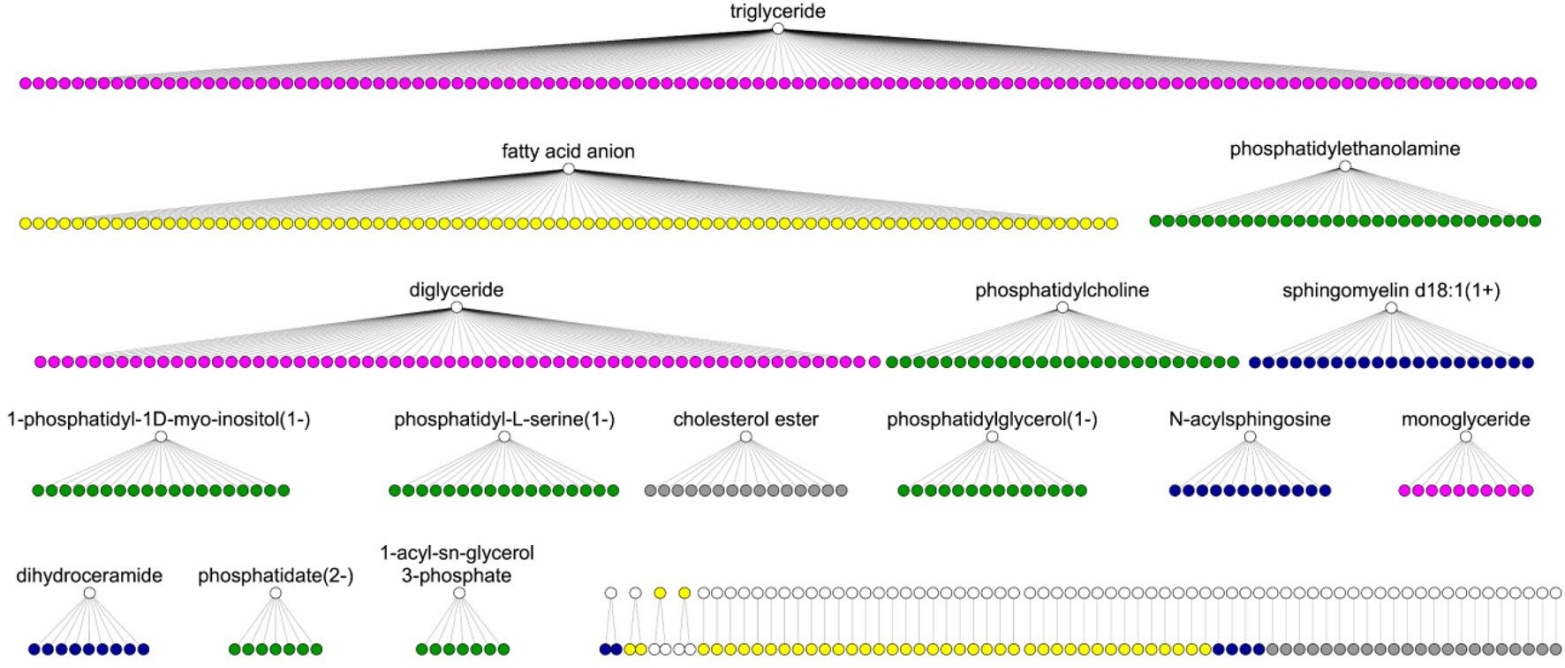
MetaboHUB chemical library matching. White nodes correspond to lipids (species or classes) in Recon2.2 metabolic network and colored nodes correspond to lipids in the MetaboHUB library. Two nodes are connected if there is a match between the lipid library and the network node. Colors depict main lipid classes: fatty acids (yellow), glycerophospholipids (green), sphingolipids (blue), sterol lipids (grey) and glycerolipids (pink)

79% of the fatty acids can be matched on Recon2.2, but with a large variability in the precision of the matching: only 21% of them have an exact match (or with an acid–base relation) on the network, whereas a large proportion match with the parent “Rtotal” which corresponds to the generic fatty acid anion. A high percentage of matching does not necessarily reflect an accurate representation of the lipid class in the network, as several different metabolites from the library may match to the same parent metabolite in the network (Fig. 4). For instance, although 100% of the 189 glycerolipids with ChEBI identifiers have matches in Recon2.2, they match on only 3 different metabolites (“mag_hs”, “dag_hs” and “tag_hs”). Similarly, 74 out of the 102 oxylipins with ChEBI identifiers match on the fatty acid anion in Recon2.2 (Fig. 4).

A few lipids, mainly unsaturated fatty acids and sterol lipids, have a negative matching distance, meaning that a more precisely defined species is present in the network. These cases correspond to stereoisomers that cannot be distinguished experimentally but that are represented by two distinct metabolites in the network.

### Class mapping of the NASH dataset

**Fig. 5:**
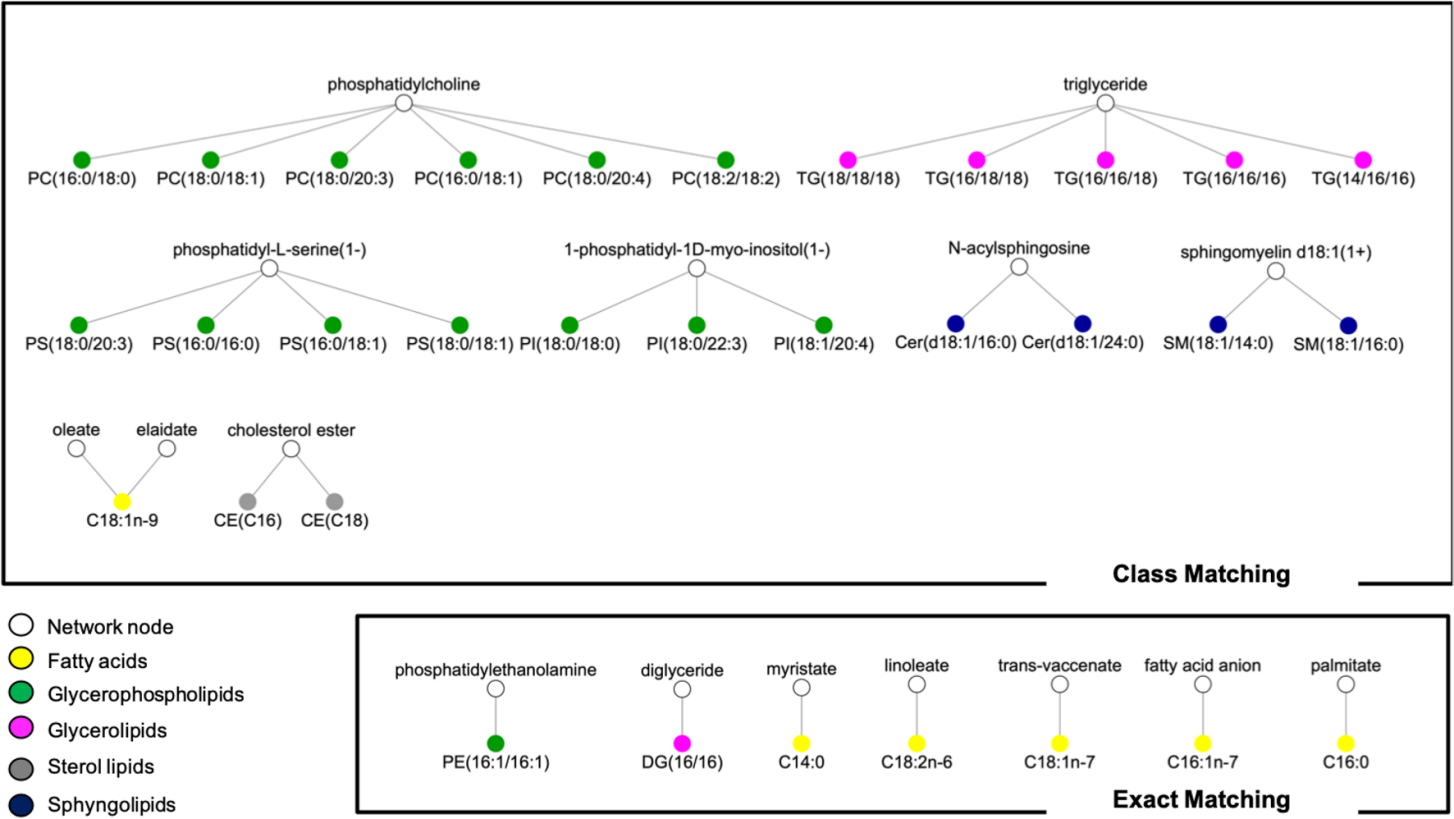
NASH lipidomics dataset matching on Recon2.2. White nodes correspond to lipids (species or classes) in Recon2.2 metabolic network and colored nodes correspond to lipids in the NASH lipidomics fingerprint. Two nodes are connected if there is a match between the lipid library and the network node. Colors depict main lipid families: fatty acids (yellow), glycerophospholipids (green), sphingolipids (blue), sterol lipids (grey) and glycerolipids (pink)

A NASH lipidomic signature, consisting in 32 lipids discriminating NASH patients from healthy subjects and NAFLD patients, was mapped onto to the metabolic network to identify which metabolic pathways are mainly involved in the development of the disease. Using our exact and class matching approach, we were able to match all the 32 lipids from the signature with network lipids. An exact matching was obtained for most fatty acids, whereas all the phosphatidylcholine, phosphatidylserine, phosphatidylinositol and triglyceride compounds were each matched with their general class compound (Fig. 5).

**Fig. 6:**
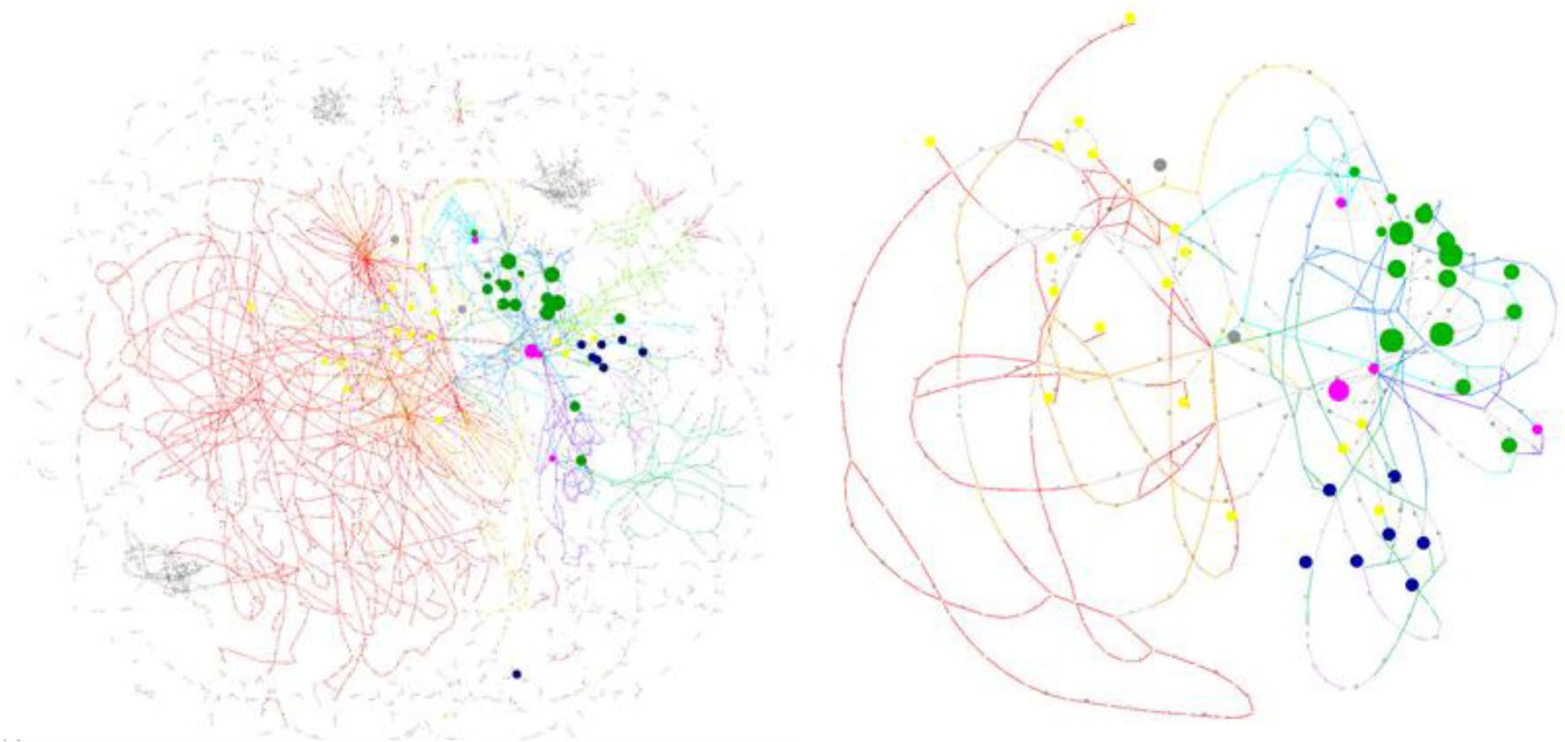
Mapping of NASH signature metabolites onto Recon2.2 (**a**) and subnetwork of Recon2.2 reactions connecting these metabolites (**b**). Main pathways are colored: red for fatty acid oxidation, orange for fatty acid synthesis, dark blue for glycerophospholipid metabolism, pink for glycosphingolipid metabolism, purple for inositol phosphate metabolism, dark pink for linoleate metabolism, light blue for phosphatidylinositolphosphate metabolism, dark green for Sphingolipid metabolism and light green for vitamin A metabolism. The mapped metabolites are colored depending on their lipid family: yellow for fatty acyls, green for glycerophospholipids, blue for sphingolipids, pink for glycerolipids and grey for sterol lipids. The size of the node corresponds to the number of lipids from the NASH dataset that are mapped onto each network metabolite (between 1 and 6). Subnetwork extraction and visualization were performed with MetExplore

Mapping the metabolites onto the metabolic network and computationally extracting a subnetwork allows to identify which metabolic reactions connect the metabolites from the signature and therefore are more likely to be modulated in the development of the disease (Fig. 6). As expected and as pointed out by Chiappini et al., these metabolic reactions mostly consist in fatty acid synthesis and oxidation reactions. Many phosphatase and phosphotransferase reactions, belonging to the glycerophospholipid metabolism pathway are also highlighted in the subnetwork corresponding to the NASH fingerprint. Locating the lipids from the NASH lipidomic signature within the global metabolic network and identifying the metabolic subnetwork associated with the signature provide some clues and hypotheses for the interpretation of the metabolic processes involved in the development of the NASH disease.

## Discussion

Ontology creation and curation are mainly a manual process. The main consequence is that some parts of the ontology may be more or less detailed. This heterogeneity has an impact on the distances used in our method to retrieve the right mapping in the ontology. Ideally, it is expected that chemical similarity/relatedness between two nodes will be proportional to the length of the paths between the nodes in the hierarchy. But in practice it is not always the case. For instance, for oxylipins, we found no matches for 9,10-DiHODE in the network whereas 12,13-DiHOME matches with the generic compound “Fatty acid anion”, although both of them are obviously fatty acids. The non-matching for 9,10 DiHODE results from a very poor detailed branch in the ontology of this compound which is directly related to the global class “lipids” whereas the branch including the 12,13-DiHOME is more detailed, going through “DiHOME”, “monounsaturated fatty acid”, “unsaturated fatty acid” to “fatty acid”. Nevertheless, efforts toward aggregating information on lipids is ongoing in ChEBI, LM or Swiss lipids.

Other ontologies exist to describe lipids, such as LM. However, LM ontology misses some layer of identification/annotation: for instance, it takes into account only the completely described molecular species for complex lipids (such as PC(18:0/18:1)), which cannot always be experimentally accessed, whereas the more generic entities such as PC(36:1), which can be measured, are not described in the ontology. Choosing a specific ontology also has some impact when some metabolites are not available in the database. For instance, in MetaboHUB lipid library, manual curation did not allow to find ChEBI for 259 lipids. This list had been sent to ChEBI administrators.

The choice of the database to use for identifier matching should be driven by the annotations present in the network and experimental dataset: in the case of Recon2.2, more metabolites were annotated with ChEBI identifiers, than with LM identifiers for instance, which is expected as the LM database only covers the lipids species in contrary to ChEBI which is more generic. Therefore, one other advantage of using the ChEBI ontology is that the developed methodology can be alternatively used for other metabolomic data sets beside lipids. Inversely, our ontology-based matching being a generic approach, it can easily be adapted and applied to any other ontology of interest. Similarly, this approach can also be applied to any metabolic network containing a sufficient number of ChEBI annotated metabolites. A specific effort has been made in Recon2.2 to increase the annotation of metabolites with ChEBI identifiers, but this is not the case for all networks: for instance, although Recon3D (Brunk et al. 2018) includes more lipids compounds than Recon2.2, the number of ChEBI currently annotated metabolites is similar to Recon2.2.

A large range of tools and web servers is available to perform identifier conversion or identifier search based on names (BridgeDB, CTS, HMDB, Metabox, SwissLipids id mapper …). Nevertheless, all these solutions are not oriented toward GSMN mapping (apart from KEGG identifiers, but which are partly covering Recon2.2). The most advanced solution to perform mapping between molecule names and network elements is MetaNetX server. The matching allows to make connection with networks from KEGG, SEED, BioCyc, Bigg (which includes Recon networks), HMR and from Maranas lab. The main difference resides in the method used to perform this matching. In MetaNetX, only molecular structure is used to identify the most chemically similar element in the network. Nevertheless, the result of such matching does not provide the reasons leading to a match while ontology-based matching will provide the path connecting metabolites. Moreover, it allows multiple matches when there is a possible ambiguity. For instance, searching in MetaNetX for octadec-9-enoic acid, ChEBI: 36021 returns one metabolite (9E)-octadecenoate (ChEBI: 30825) hence missing oleate (ChEBI: 30823) as shown in Fig. 2. One of the possible future directions would be to combine both approaches.

Results presented in this article show that using the ontology-based matching often result in mapping several lipids from the experimental dataset onto the same node in the network. This can lead to difficulties in the interpretation because, even if these molecules belong to the same family, the concentrations of these different molecular species can be different. This points out that metabolic network models, or some parts of them, may not be detailed enough to allow for the interpretation of lipidomics dataset. When variations in specific molecular species are of biological relevance, one way to circumvent the issue would be to enhance the details of the corresponding metabolic pathways in the network to get a more comprehensive description and visualization of all the reactions involved. Some attempts have been made in that sense, but the resulting network can very rapidly increase in size and become very large (Smallbone 2013).

Beyond mapping of data in GSMN, matching identifiers is instrumental in integrating data produced by different analytical platforms in order to go further in metabolome coverage [see the approach implemented in MetaMaks (Redestig et al. 2010)]. The proposed method could also be of interest to tackle this challenge.

## Conclusion

The ontology-based method proposed in this article to map lipids onto metabolic networks is a step forward in the integration of metabolic profiling data in the context of metabolic networks. By using ontology hierarchical classification, we demonstrate that we can improve and facilitate the link between GSMN and a chemical library or a lipidomics dataset. In addition, the approach provides ways of improvements in terms of network curation (which parts of the network need to be expended) and in terms of lipidomics data annotation. It is also important to note that the same ontology-based mapping can be applied to all metabolites since ChEBI aims at describing all small molecules. The method is for now only focused on ChEBI identifiers but it could be extended to other identifiers as long as they are associated with an ontology. The implementation of the method is publicly and freely available in an open source python library and is also accessible through MetExplore web server.

One future direction would be to associate ontology based mapping and chemical based mapping. To do so both metabolomics and network modelling communities need to annotate metabolites with both ontology identifiers (e.g. ChEBI) and chemical representations (InChIKeys, SMILES).

## Electronic supplementary material

Below is the link to the electronic supplementary material.

Supplementary file1 (DOCX 9797 kb)

Supplementary file2 (XLSX 17 kb)

Supplementary file3 (XLSX 135 kb)

Supplementary file4 (XLSX 80 kb)

Supplementary file5 (XLSX 53 kb)
