# Supplementary material for: Improving lipid mapping in Genome Scale Metabolic Networks using ontologies
Source: Metabolomics. 2020 Mar 25;16(4):44. doi: 10.1007/s11306-020-01663-5 (PMC7096385; doi:10.1007/s11306-020-01663-5)
Supplement: Supplementary file 1 — Supplementary file1 (DOCX 9797 kb) [file 11306_2020_1663_MOESM1_ESM.docx]

| **MetExplore**:  Omics data analysis in the context of metabolic networks |
| --- |
| 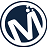 |
| **Nathalie POUPIN**  **Florence VINSON**  **Maxime CHAZALVIEL**  **Ludovic COTTRET**  **Clément FRAINAY**  **Fabien JOURDAN** |

Omics data analysis in the context of metabolic networks


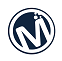


[www.metexplore.fr](http://www.metexplore.fr)

twitter: @metexplore

contact: contact-metexplore@inra.fr

[**Introduction** 3](#_30j0zll)

[**Practice 1.**](#_3znysh7) **Exploring metabolic networks with MetExplore** 5

[**1.1.**](#_2et92p0) **Selecting a BioSource 5**

[∙](#_tyjcwt) BioSource search strategy based on grouping 5

[∙](#_3dy6vkm) BioSource search strategy based on field search. 6

[∙](#_1t3h5sf) BioSource search strategy using “Selected BioSource” search (right panel) 6

[∙](#_4d34og8) Selecting a BioSource 7

[**1.2.**](#_2s8eyo1) **Exploring the content of metabolic networks 8**

[∙](#_17dp8vu) Find information about metabolites, reactions … 9

[∙](#_3rdcrjn) Explore associations between genes, reactions and metabolites using the “filter” feature 9

[**Practice 2.**](#_26in1rg) **Mapping of lipidomics data** 11

[**2.1.**](#_lnxbz9) **Finding the relevant database identifiers 11**

[∙](#_35nkun2) Search identifiers for the NASH dataset metabolites using CTS 11

[∙](#_1ksv4uv) Search identifiers for the NASH dataset metabolites using ChEBI and LIPID MAPS database: 12

[**2.2.**](#_44sinio) **Matching dataset identifiers with metabolic network identifiers 14**

[**2.3.**](#_2jxsxqh) **mapping obtained IDs 20**

[∙](#_z337ya) Getting the list of network metabolites to map 20

[∙](#_1y810tw) Performing mapping in MetExplore 21

[**2.4.**](#_4i7ojhp) **Identifying pathways and reactions related to the mapped data 22**

[**Practice 3.**](#_2xcytpi) **Visualizing mapped data and subnetworks of interest** 25

[**3.1.**](#_1ci93xb) **Creating a subnetwork of interest based on pathways 25**

[**3.2.**](#_3whwml4) **Visualizing the selected subnetwork 26**

[∙](#_2bn6wsx) Global visualization 26

[∙](#_qsh70q) Highlighting cellular compartments and metabolic pathways 27

[∙](#_3as4poj) Duplication of side compounds 29

[**3.3.**](#_49x2ik5) **Highlighting mapped data 30**

[**References 33**](#_2p2csry)

**Introduction**

MetExplore is a web server dedicated to the analysis of genome scale metabolic networks, with a special care taken to allow analyzing metabolomics data in the context of these networks.

## MetExplore story

MetExplore project was launched in 2009 by Ludovic Cottret, Florence Vinson and Fabien Jourdan. This original work included several collaborators from France (INRA, INRIA) and University of Glasgow (UK). It was first published in Nucleic Acids Research in 2010.

Since that date, MetExplore had been extended with new features and the server is now hosted on a stable and sizable computational infrastructure (provided by Toulouse Bioinformatics Facility).

MetExplore latest version had been published in NAR in 2018 (see “how to cite” section).

## How to cite MetExplore

Cottret,L. *et al.* (2018) MetExplore: collaborative edition and exploration of metabolic networks. *Nucleic Acids Res.*, **46**, W495–W502.

## Following and getting in touch

We try to provide feedbacks as much as possible via different media:

- **website:** [**www.metexplore.fr**](http://www.metexplore.fr) This website is the frontend of MetExplore. It provides news on training, publications, documentation and the crew.
- **Mail:** [**contact-metexplore@inra.fr**](mailto:contact-metexplore@inra.fr) This emails reaches the whole MetExplore crew. We aim at answering as fast as possible to questions, bugs … etc.
- **Documentation:** [**https://metexplore.toulouse.inra.fr/metexplore-doc/index.php**](https://metexplore.toulouse.inra.fr/metexplore-doc/index.php) This page gathers documentations for the different MetExplore features.
- **Twitter**: **@MetExplore** for regular updates and news about social and scientific life of the crew.

## This document

The aim of this document is to guide you through the use of MetExplore web server. It will not cover all the functionalities MetExplore can achieve but it will provide you with the basic concepts and functions. **The present document and practical focuses on lipidomics data mapping and visualization.**

To ease the use of the document, *practical parts and tasks to do are displayed in grey and introduced by ⮚*, and **definitions of concepts specific to MetExplore software are displayed in blue**.

## Dataset

In this tutorial, we will use a **lipidomics dataset from the paper of Chiappini F. *et al*.** (Metabolism dysregulation induces a specific lipid signature of nonalcoholic steatohepatitis in patients. Sci Rep. 2017;7: 46658. [1]).

Briefly, in this study, lipidomics analyses were performed on liver biopsies from healthy patients and from patients with non-alcoholic fatty liver (NAFL) and non-alcoholic steatohepatitis (NASH) using gas-phase or liquid-phase chromatography coupled with to mass spectrometry. A random forest-based machine learning approach was used and allowed to identify a signature of 32 lipids, discriminating between healthy livers, NASH and 3 grades of NAFL. **This list of 32 discriminating lipids will be used as an example dataset in this tutorial**.

As these data were obtained on human samples, we will work on the **human genome-scale metabolic network Recon2.2** [2]. We will exemplify grid usage and exploration of network elements using this network.

All data and inputs needed for this tutorial are provided in supplementary file 5.

## To start using MetExplore

- *Go to the* ***MetExplore homepage*** *(*[*http://www.metexplore.fr*](http://www.metexplore.fr)*) and click on the "****START MetExplore****" button.*
- *From this home page, you can access the MetExplore documentation, the MetExploreViz documentation, the MetExplore webservices and the registration page.*

**Note that it is better to use Chrome web browser.**


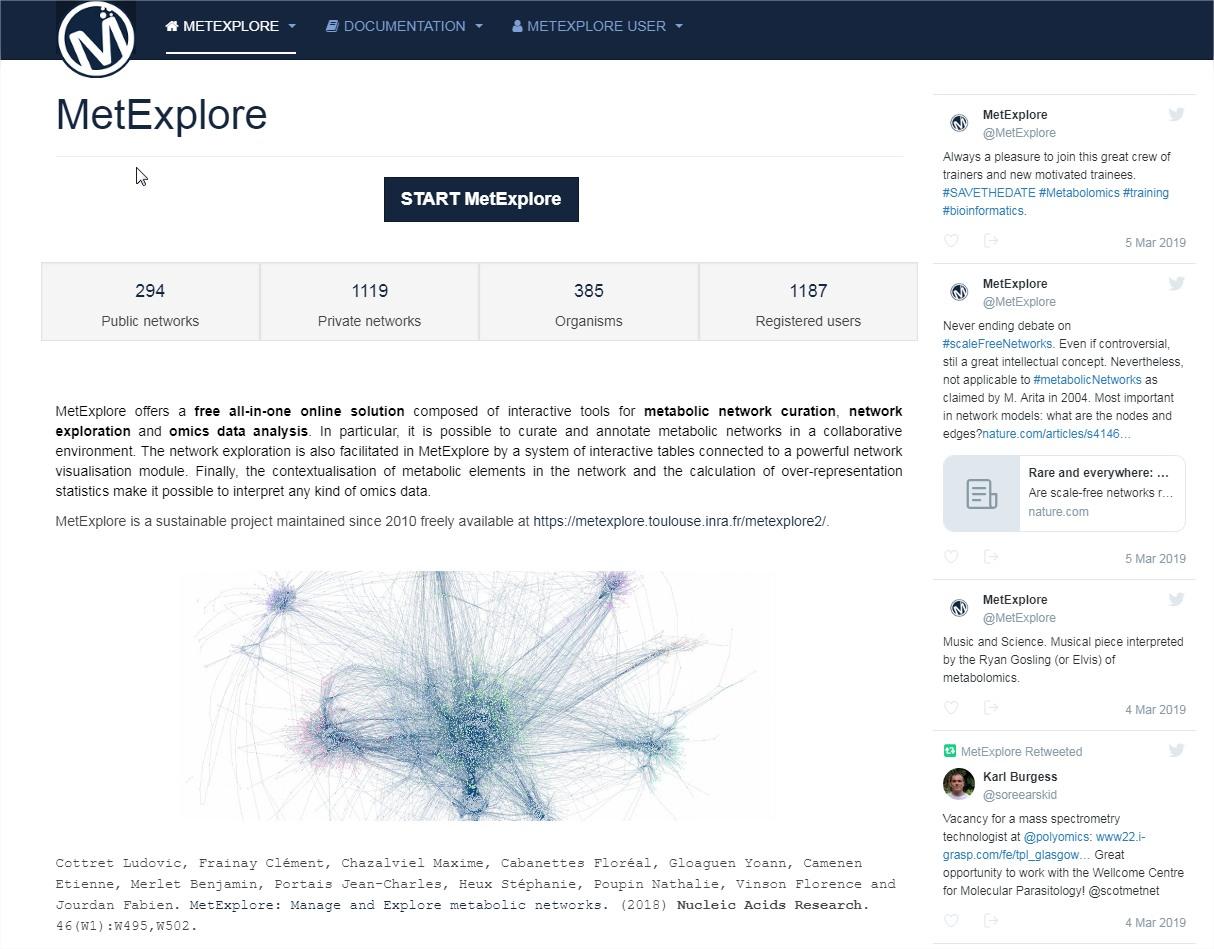


1. *MetExplore homepage*
2. **Exploring metabolic networks with MetExplore**

## Objectives: The aim of this section is to describe how you can explore the content of a metabolic network and get familiar with the MetExplore interface.

# Selecting a BioSource

### What is a BioSource?

Several genome scale metabolic networks can be available for a given organism. In MetExplore, we use the term “**BioSource**” to refer to a metabolic network. The aim is to avoid confusion and keep the flexibility of having several networks for several organisms. Hence, a BioSource will have a MetExplore specific identifier called “**Biosource id**”.

MetExplore is providing access to networks imported from public databases (BioCyc https://biocyc.org/[3,4], KEGG [5], BioModels [6,7] …) and from SBML files [8,9]. All these files are present in the "public BioSource" repository. Note that you can also register and upload your own SBML files. You will then be the only one to be able to access these networks and will have the opportunity to share them with others.

BioSources are classified using the following fields:

- **Organism**: all BioSources corresponding to the same organism.
- **Source Database**: the database hosting the BioSource (e.g. Biomodels, MicroCyc …)
- **Database Type**: the generic provenance of a BioSource. It can for now be only of three kinds: KEGG, BioCyc or SBML.
- **Status**: kind of visibility of a BioSource. Public means that the BioSource is accessible to anyone using MetExplore. Private means that the BioSource is owned by someone registered in MetExplore. This BioSource can be private but also shared with a selection of collaborators.
- **Projects**: when you are the owner of several BioSources, you can group them in projects (e.g. all your human metabolic networks).

**As data from our example dataset have been obtained on human liver biopsies, we will focus on the human genome-scale metabolic network.** There are 12 public metabolic networks for *Homo sapiens* in MetExplore. These biosources are either imported from SBML files provided in an article or issued from different databases: KEGG, BioCyc and specific databases for human models such as VMH (Virtual Metabolic Human, <https://www.vmh.life/>), HMA (Human Metabolic Atlas, <http://www.metabolicatlas.com/>). Note that some of the biosources available for *Homo sapiens* are tissue specific (as specified in “Strain”).

In this practical, we will use the **human metabolic network Recon2.2**, which has been published in 2016 by Swainston *et al.* [2] and is an update (partly manually curated) of the Recon2 metabolic network published by Thiele *et al*. in 2013 [10].

**The first thing to do before starting working on a metabolic network is to select the right BioSource. In MetExplore, this task can be achieved in different ways.**

- **BioSource search strategy based on grouping**

By default, the grouping is done using organisms. You can change the way BioSources are classified in the BioSource panel.

- *To do so, change the “Group by” field and select one of the grouping options: Organism, Source Database, Database Type, Status or Projects.*


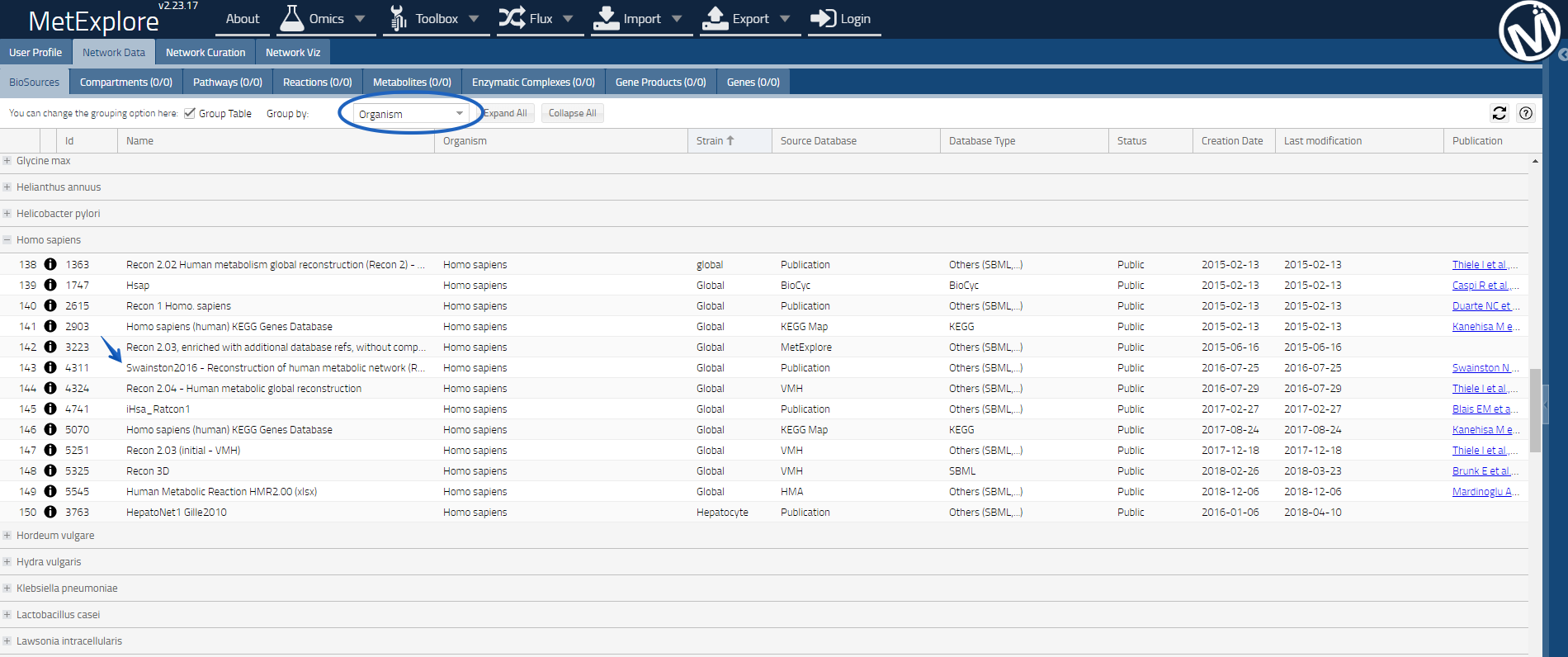


1. *MetExplore BioSources grid grouped by organism*

You can remove the grouping by unchecking the “Group by” check box.

- **BioSource search strategy based on field search.**

By clicking on the arrow at the top of each column, you can perform a search.

- *Click on the arrow next to Organism and it will start filtering the column.*


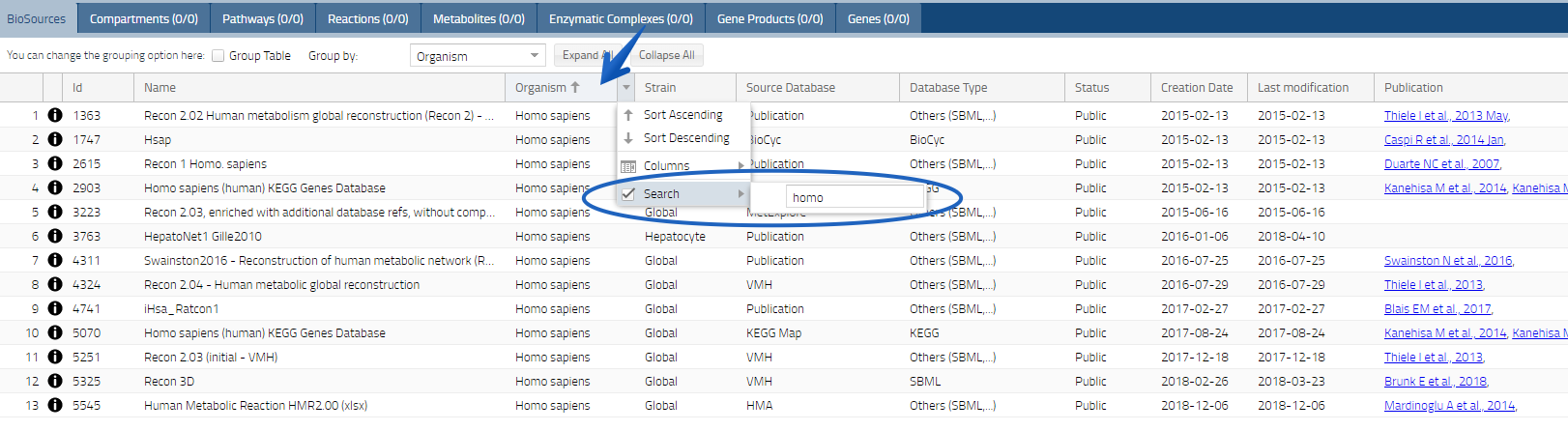


1. *Selection of a BioSource by searching a specific organism in MetExplore*

- **BioSource search strategy using “Selected BioSource” search (right panel)**

The right panel of MetExplore contains BioSource information.

You can use the combo box to search BioSources.


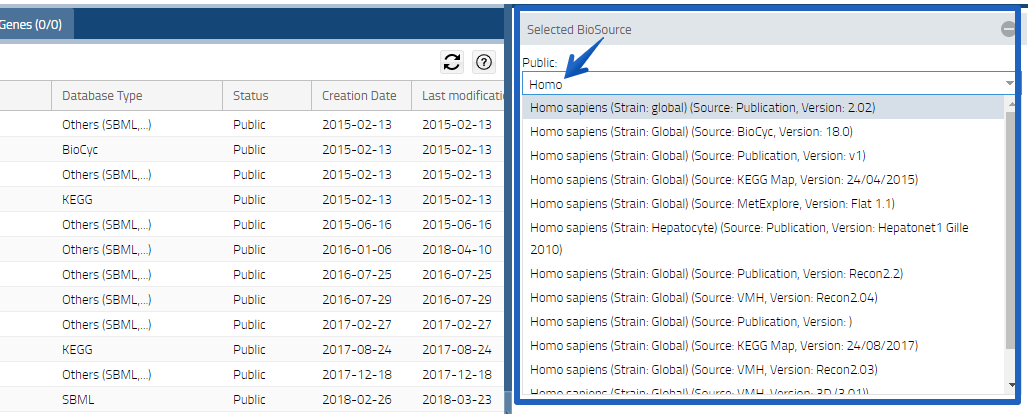


1. *Selection of a BioSource using the right panel in MetExplore*

- **Selecting a BioSource**

Once you found your BioSource of interest in the list. You have to select it to load all the content of the BioSource and start working on it.

To do so you can:

- *Right click on the line and click on “Select BioSource”*

or

- *Double click on the line*


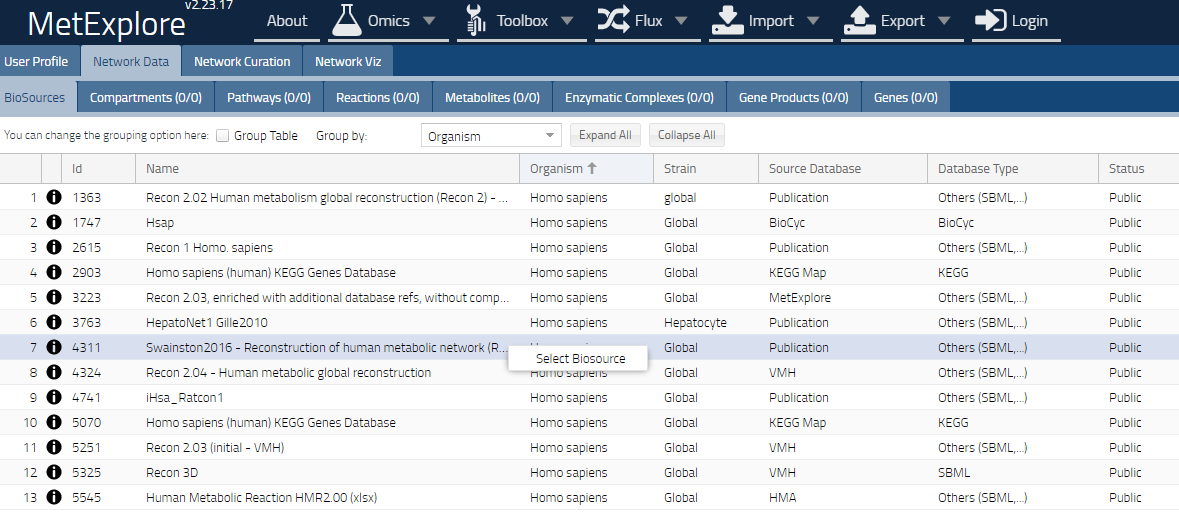


1. *BioSource selection in the main panel in MetExplore*

- *Select the BioSource corresponding to the human metabolic network Recon2.2 (#4311)*

Once a BioSource is selected, all the panels will be populated with the number of elements displayed.


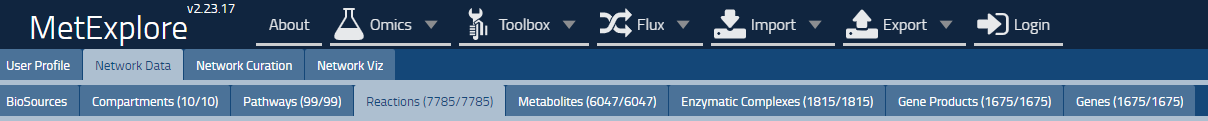


Moreover, the right panel will contain all information related to the BioSource.


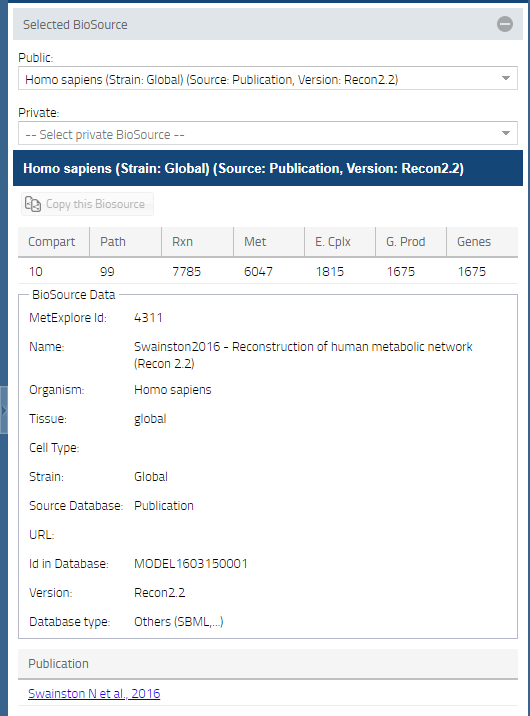


1. *Information on selected BioSource displayed in the right panel in MetExplore*

# Exploring the content of metabolic networks

The content of the metabolic network are displayed in grids, under the “Network Data” tab.

### What is a “Grid”?

The concept of **“grid”** in MetExplore corresponds to a spreadsheet like representation of the content of the various sets used to describe a metabolic network. Hence, there will be a grid for cellular compartments (“**Compartments**”), metabolic pathways (“**Pathways**”), reactions (“**Reactions**”), metabolites (“**Metabolites**”) up to genes (“**Genes**”).

Grid come with several features (e.g. filters) and are editable if you are working on you own private BioSource.


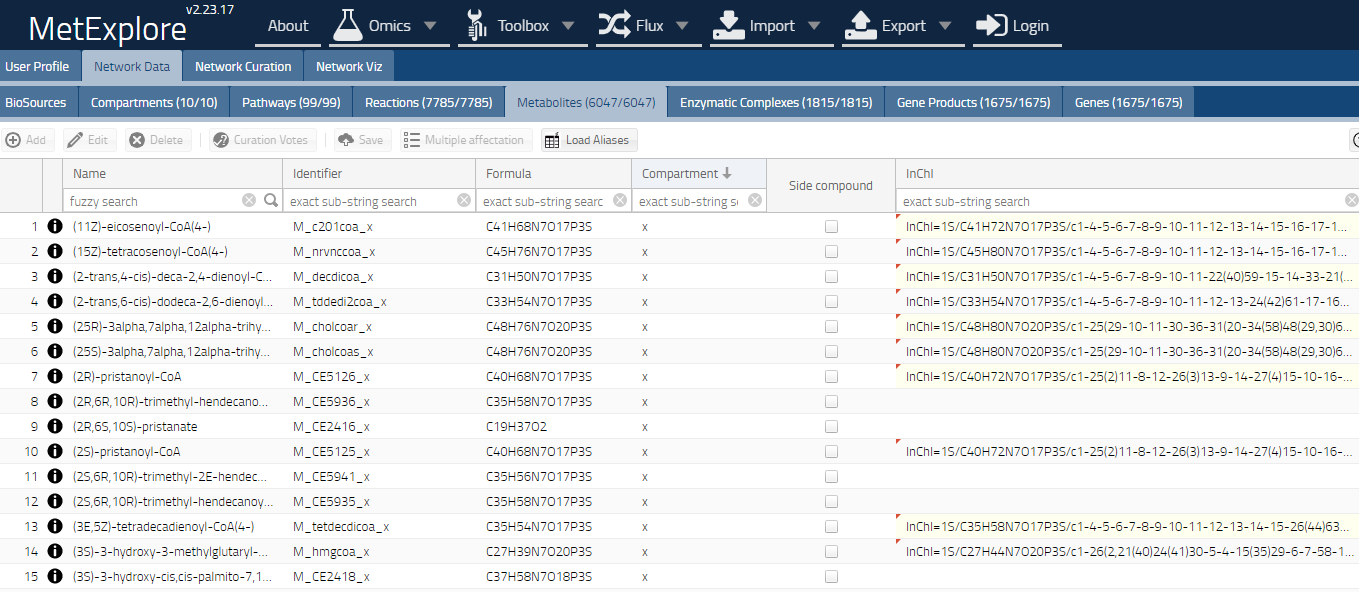


1. *MetExplore representation of metabolites network content*

The columns displayed on each grid can be rearranged (reordered, removed, added) and sorted by clicking on the right side of each column header to show up the column menu.

Data in each column can also be searched for, by using the **"Search"** field". The data in the table will be filtered depending on the values contained in this column. Note that this filter is not propagated to the other grids (unlike with the “filter” option, see next section).

**
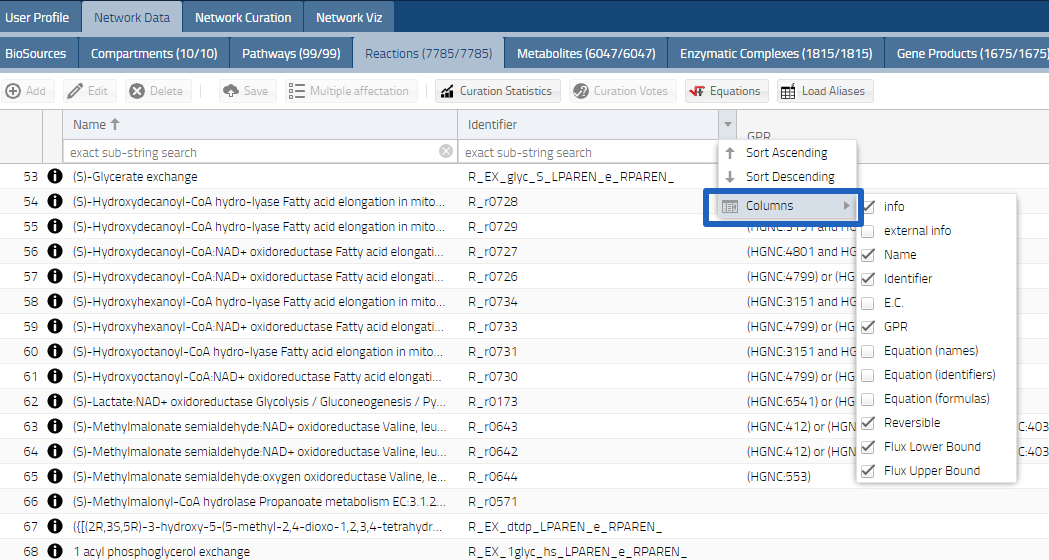
**

1. *Displaying and arranging grid columns in MetExplore*

- **Find information about metabolites, reactions …**

Information about each network element (metabolite, reaction, gene …) can be accessed by clicking on the
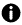
 symbol next to the element name. This will open a popup window, containing information about:

- **for reactions**: equation, associated genes and associated pathways


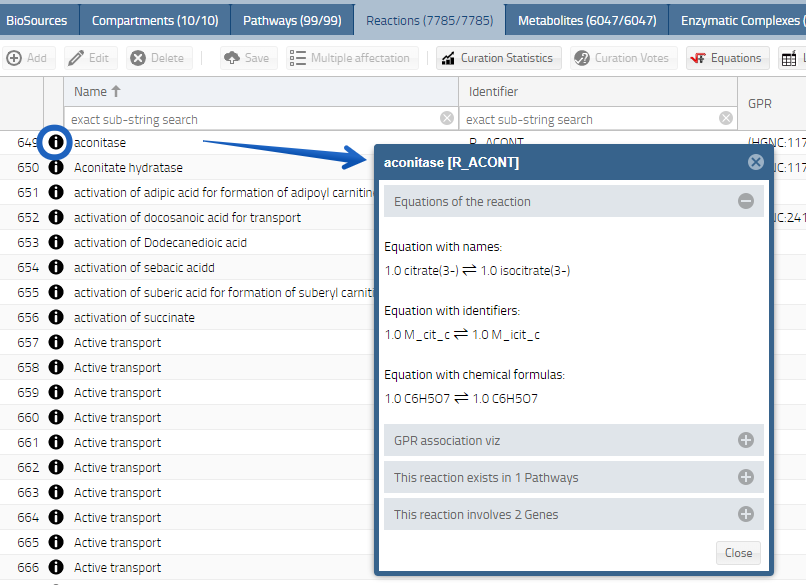


1. *Reaction detailed information popup in MetExplore*

- **for metabolites**: the identifiers from different databases (if available in the initial SBML)


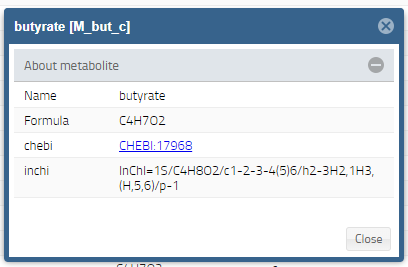


1. *Metabolite detailed information popup in MetExplore*

- **Explore associations between genes, reactions and metabolites using the “filter” feature**

One of the main features of MetExplore is the ability to filter data grids from another data grid.

The **“filter”** concept: all grids in MetExplore (e.g. metabolites, reactions …) are dynamically connected. Meaning that interactions on one grid can affect the content of other grids. The central notion for this kind of interaction is the notion of **“filter”**. **A filter on a line or a set of lines in one grid will imply a selection on the other grids.** For instance, by filtering on a Pathway, one can select all the reactions corresponding to this pathway and see them displayed in the Reactions grid. Similarly, filtering on one reaction in the “Reaction” grid will select the elements corresponding to this reaction in the other grids: metabolites substrates and products of the reaction, genes required to encode the enzyme catalyzing the reaction....

Note that filters can be applied to a selection of several elements, i.e., a list of metabolites or reactions for instance.

To **apply filter**, right click on a line or a selection of lines and select “new filter on selection”. The number of elements selected in each grid appears in the tab above the grid.

To **cancel a filter**, erase the content of the "Search" field or right-click on the grid and click on "Delete Filter & Search".


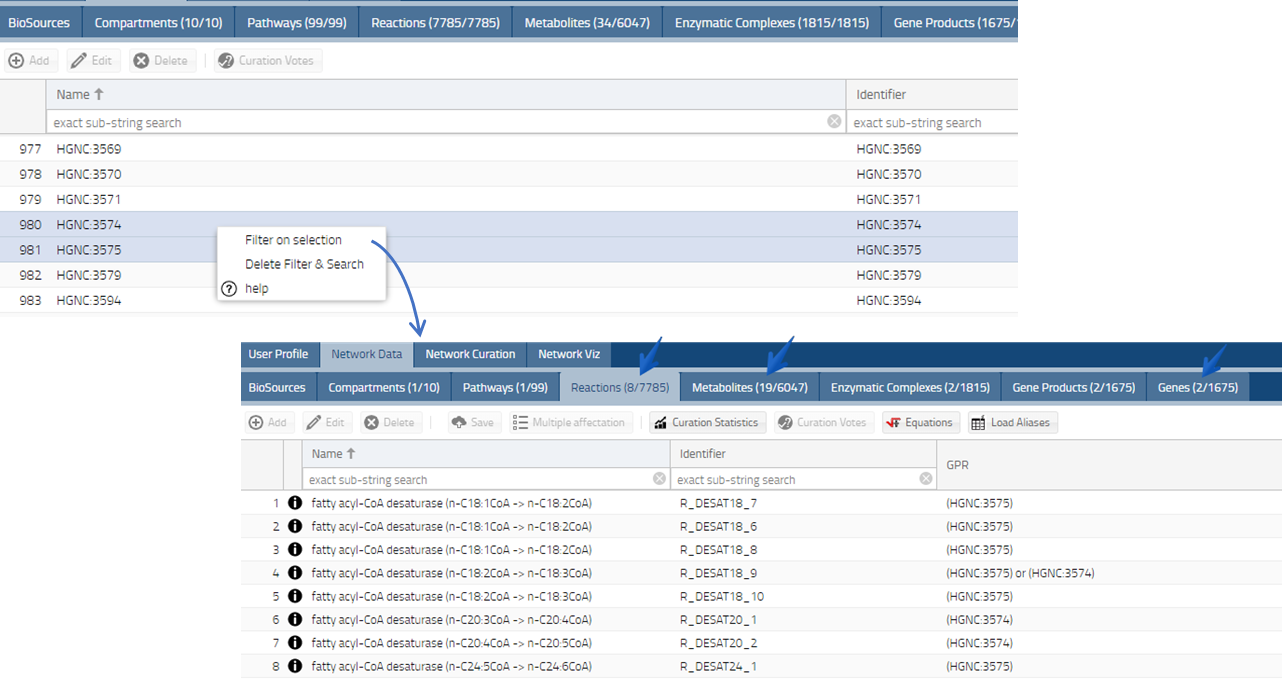


1. *MetExplore filtering on genes*

- *Find all reactions producing or consuming palmitate*


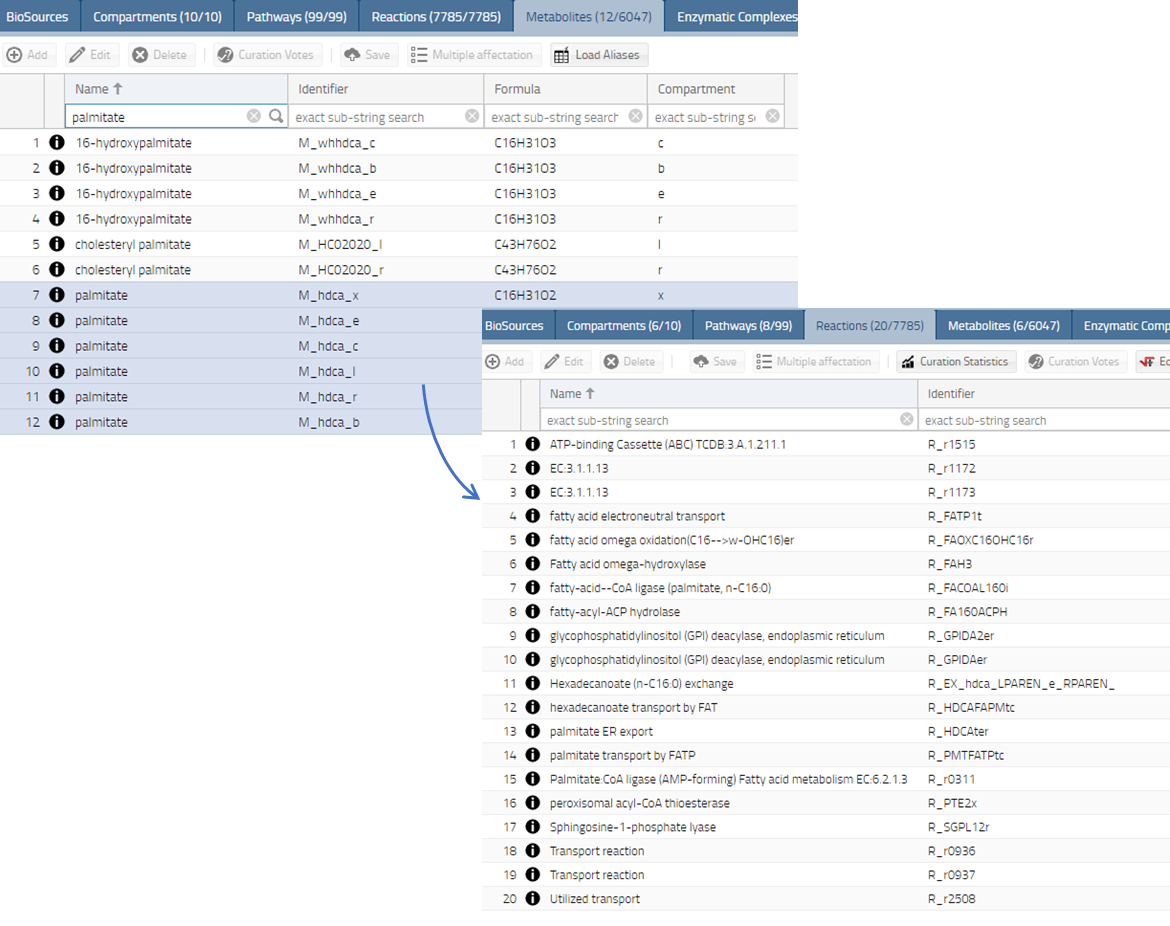


1. *MetExplore filtering on metabolites*
2. **Mapping of lipidomics data**

**Objectives**: the aim of this section is to **learn how to map lipidomics data in metabolic network**, meaning pinpointing lipids identified in experiments within metabolic networks.

As explained in introduction, we will use as a benchmark the **lipidomics signature of liver NASH evidenced by Chiappini *et al.*** [1]. The list of 32 lipids constituting this signature is provided in the “DataLipids_Chiappini2017.xlsx” file. We will thereafter refer to this list of metabolites as the **"NASH dataset"**. The challenge will be to **place these lipids within the human metabolic network, to identify potential metabolic pathways and reactions related to NASH**.

**In metabolic network reconstructions, metabolites are referenced with names and with identifiers that are specific to each network** (or each database they are issued from). For instance, in the different versions of Recon2, metabolite identifiers start by “M_” and end with a letter corresponding to the cellular compartment (e.g., “_c” for cytosol, “_m” for mitochondria …).

Metabolite names used in networks and in experimental datasets do not usually comply with any specific naming convention and come with different spellings, lower or upper-case letters… Therefore, it is often impossible or unsuccessful to perform a direct and automatic matching between metabolite names or identifiers present in the network and metabolite names provided by experimentalists.

**In metabolic networks, other identifiers issued from metabolite databases such as KEGG, CHEBI, HMDB, LipidMaps … can also be informed for each metabolite**. **These identifiers can be used to make the link between metabolites in experimental dataset and metabolites in metabolic networks**, but this requires to first find **such database identifiers for the dataset metabolites.**


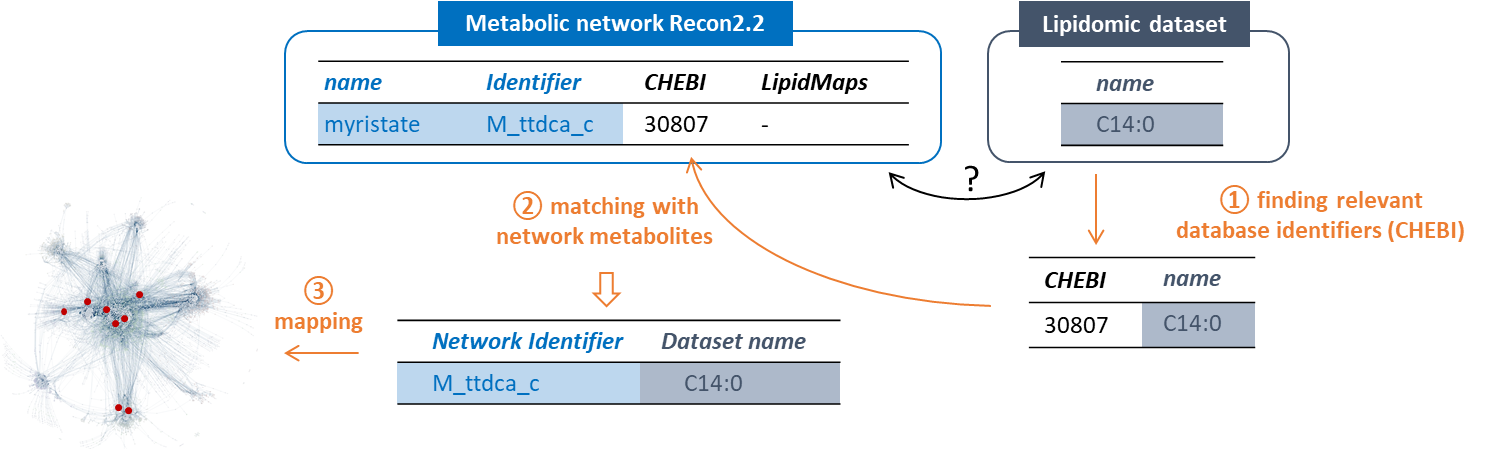


1. *How to link metabolites in experimental dataset and metabolites in metabolic networks*

To make the link between the metabolites present in the NASH dataset and the metabolites in the human metabolic network, you will therefore need to (1) **find corresponding database identifiers for the NASH dataset metabolites** and (**2) match them with the ones present in the metabolic network**.

# Finding the relevant database identifiers

**The first step consists in finding different database identifiers for the metabolites in your dataset**, by searching in the different public databases such as Lipid Maps (<https://www.lipidmaps.org/>), ChEBI (<https://www.ebi.ac.uk/chebi/>), HMDB (<http://www.hmdb.ca/>), … Conversion tools can also be useful for this purpose, for example the Chemical Translation Service (CTS, <http://cts.fiehnlab.ucdavis.edu/>) allows converting metabolite names into various identifiers.

- **Search identifiers for the NASH dataset metabolites using CTS**
- *Go to* [*http://cts.fiehnlab.ucdavis.edu/*](http://cts.fiehnlab.ucdavis.edu/)

You can do a single conversion or a conversion of several metabolite names.

- *Click on the "Batch Conversion" menu and then copy paste the list of metabolite names from the NASH dataset (“*[*DataLipids_Chiappini2017.xlsx*](https://docs.google.com/spreadsheets/d/1DhIw3A_ylYS3ex7QCfOEi1sbVeL77FKyPAgtNIoX3kk/edit#gid=73524087)*” file → “fig2_data”).*

Note that CTS does not accept "/" so that we have replaced them by "_".

- *Select the databases of interest (in our case: CHEBI and LipidMAPS) and click on "Convert"*


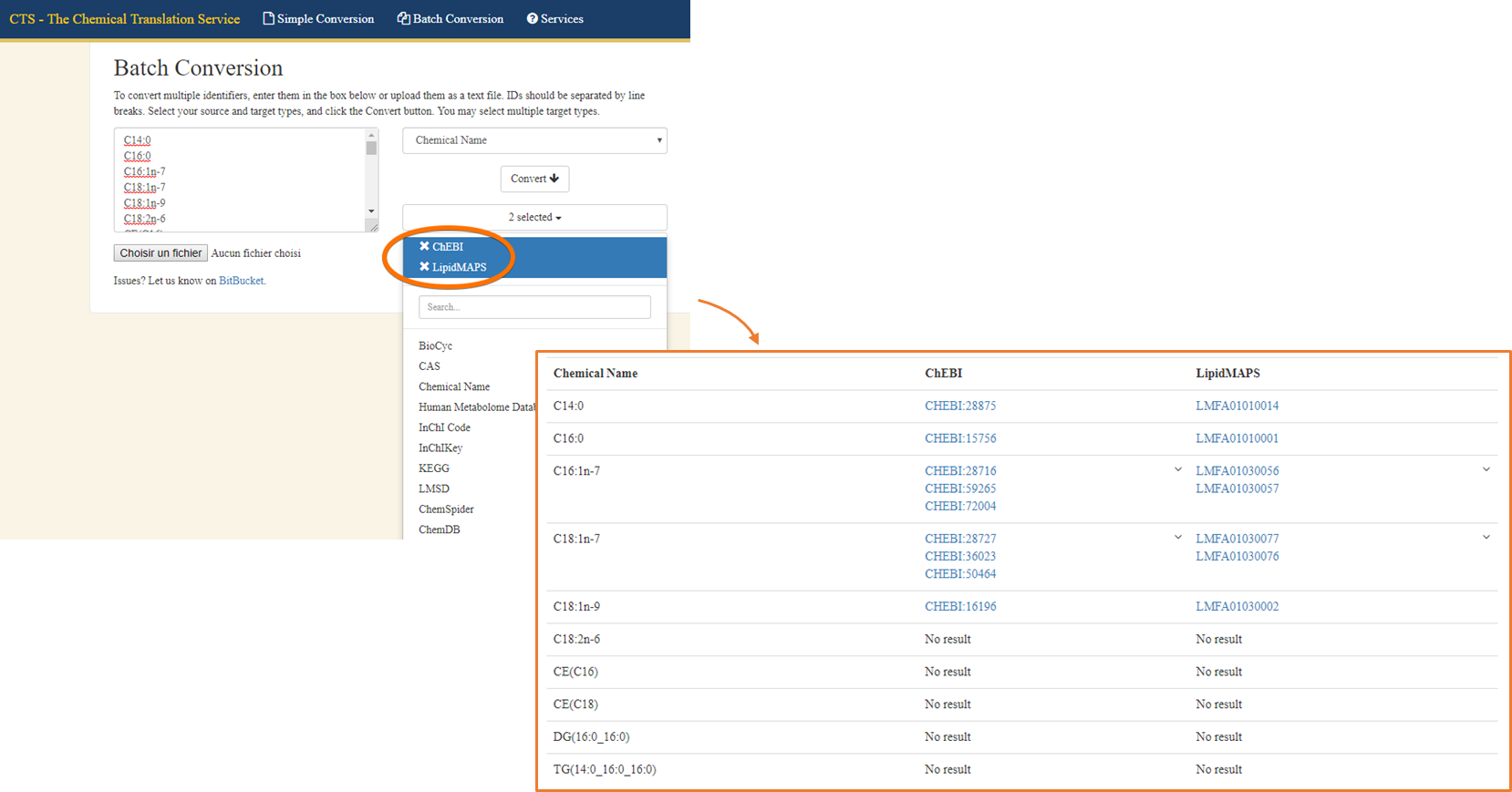


1. *Batch conversion of metabolite identifiers in CTS*

You can export the results as a CSV or TXT file.

Note that for some metabolites, there are several different possible identifiers, such as for "C16:1n-7". On the contrary, for many other metabolites, no CHEBI or LipidMAPS identifiers have been found ("not found"). For these later metabolites you need to perform a manual search in the ChEBI and LipidMAPS databases.

- **Search identifiers for the NASH dataset metabolites using ChEBI and LIPID MAPS database:**

***e.g. C18:1n-9***

- *Go to* [*https://www.ebi.ac.uk/chebi/*](https://www.ebi.ac.uk/chebi/) *and enter the name of the metabolite you are searching.*

Note that entering "C18:1n-9" does not return any results, while entering C18:1(n-9) will return 4 entries.

- *Check manually each entry to select the ones that truly correspond to the C18:1(n-9) metabolite.*

You can look at the "Synonyms" section to check for other possible names for each entry, and check that "C18:1(n-9)" is included.

You might also find some corresponding identifiers for other databases in the "Manual Xrefs" section.

"octadec-9-enoic acid" (CHEBI:36021) and "oleic acid" (CHEBI:16196) both rightly correspond to a C18:1(n-9) fatty acid, whereas "octadec-9-enoate" (CHEBI:132944) corresponds to the conjugate base and "N-oleoylphytosphingosine" (CHEBI:85204) to the C18:1(n-9) phytoceramide.


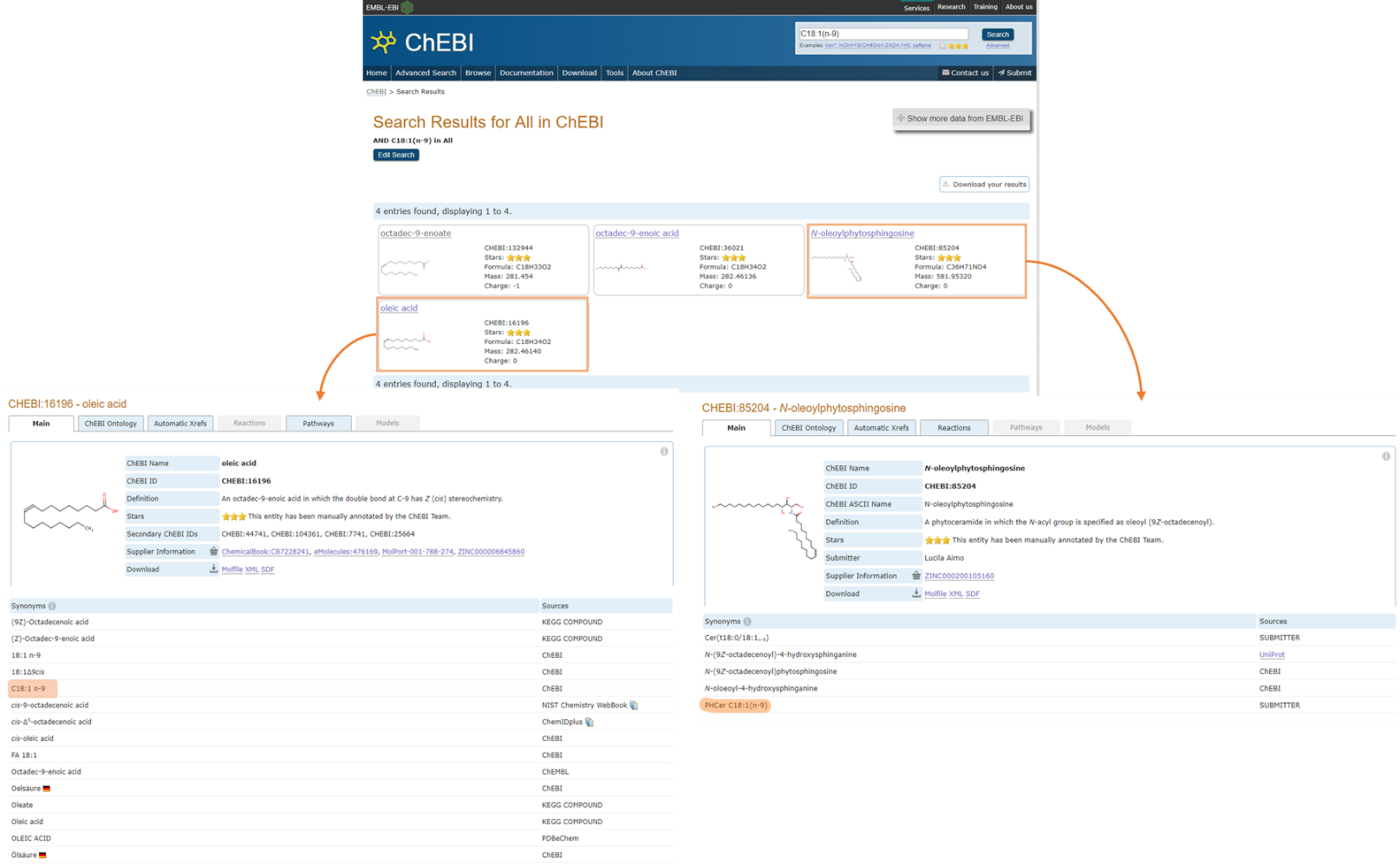


1. *Metabolite search in ChEBI*

- *Go to* [*https://www.lipidmaps.org/resources/databases/index.php*](https://www.lipidmaps.org/resources/databases/index.php) *→ Resources → Databases.*
- *Select* [*Text/ontology-based search*](https://www.lipidmaps.org/data/structure/LMSDSearch.php?Mode=SetupTextOntologySearch)
- *To search by name, enter your metabolite name in the "Name" box and click "Submit".*

Entering "C18:1n-9" will give you 2 results.

- *Click on each metabolite to get a precise description, check that it indeed corresponds to the searched metabolite and find corresponding identifiers in other databases.*


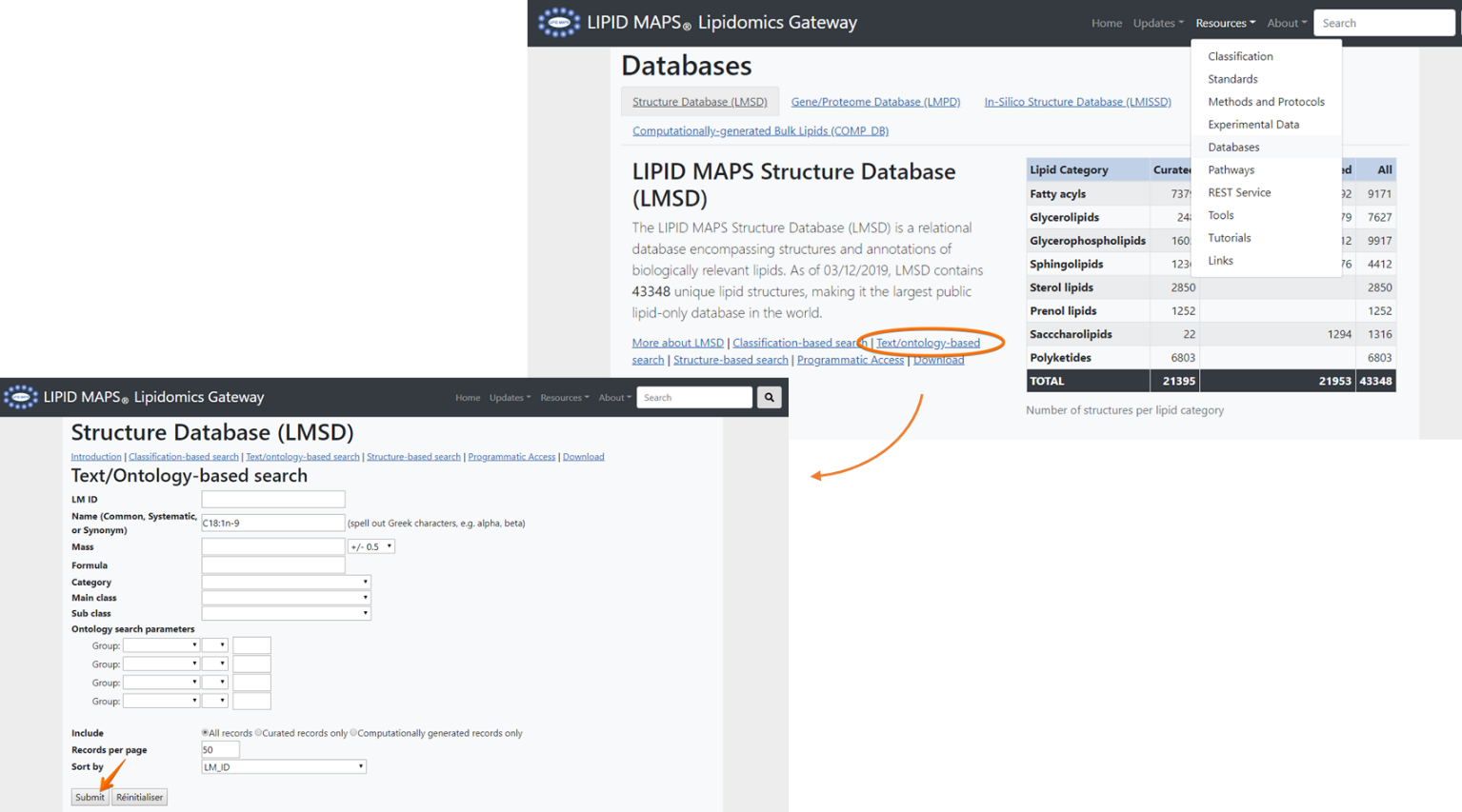


1. *Metabolites search in LipidMAPS*


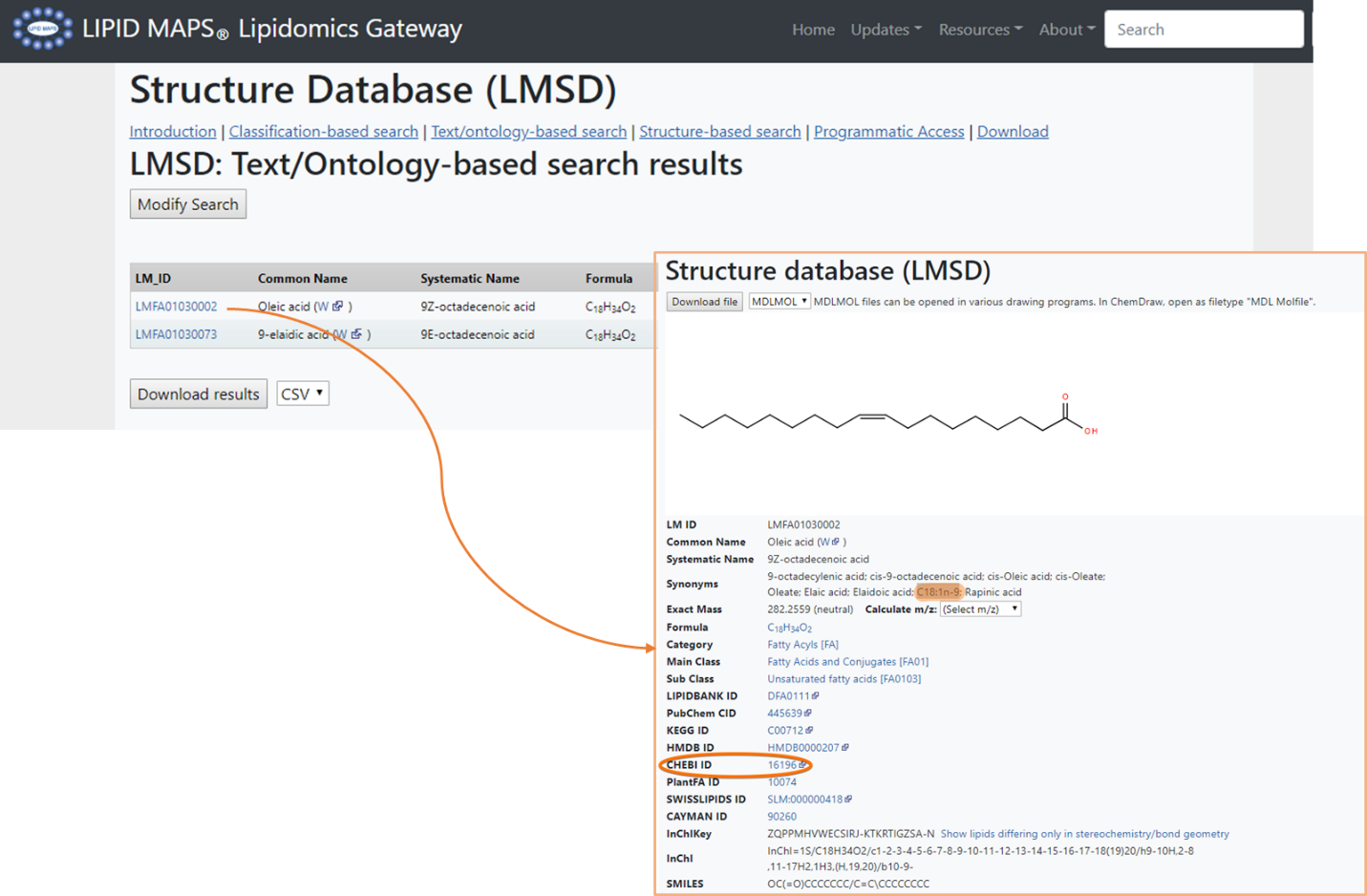


1. *Finding corresponding metabolite identifiers in other databases from LipidMAPS*

- *Retrieve CHEBI identifier(s) for C18:1(n-7) and other lipids in the NASH dataset.*

The process of finding identifiers for the metabolite dataset can be highly time consuming. **For the purpose of this tutorial a list of ChEBI identifiers corresponding to the NASH lipid dataset is provided in the “fig2_data CHEBIids” sheet of the supplementary file 5.** This list can be directly used for the “metabolite identifier matcher” tool in MetExplore.

# Matching dataset identifiers with metabolic network identifiers

To be able to map data metabolites in metabolic network, it is then necessary to find their corresponding identifiers in the network. This can be done using the “metabolite identifier matcher” tool in MetExplore. It proceeds by matching the database identifiers retrieved for data metabolites (used as input) with the ones present in the network.

In MetExplore, these identifiers can be seen by clicking on the  symbol.

Note than before doing any matching or mapping it is better to remove all filters and searches that you have done:

- *in the filtered or searched grids, right click and select “Delete Filter & Search”*
- *Select “Toolbox" → “Metabolite identifier matcher” in the menu at the top of the page: this will open a “metabolite identifier” window*


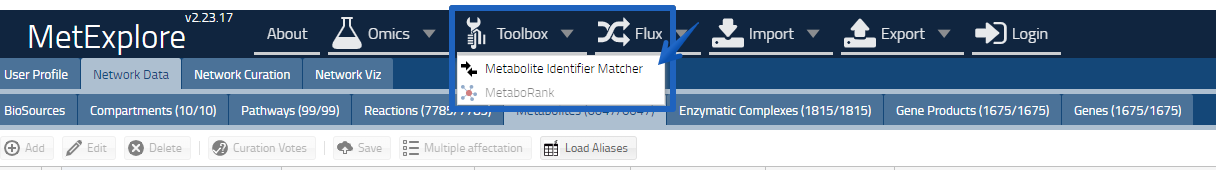


1. *Finding the network metabolite identifiers corresponding to the data metabolites in MetExplore*

- *in this window, you can simply paste your list of data metabolites with their corresponding database identifier*
- *Copy the table in the “X” sheet of the xlsx file (including the header row)*
- *Check the “consider first row of columns as headers” box to indicate that the first line of your copied dataset corresponds to the type of identifiers*
- *Do CTRL+V on the first cell of the “copy/paste in grid”*

The headers should be automatically completed. If not choose the identifier corresponding to each of your columns in the drop-down menu.


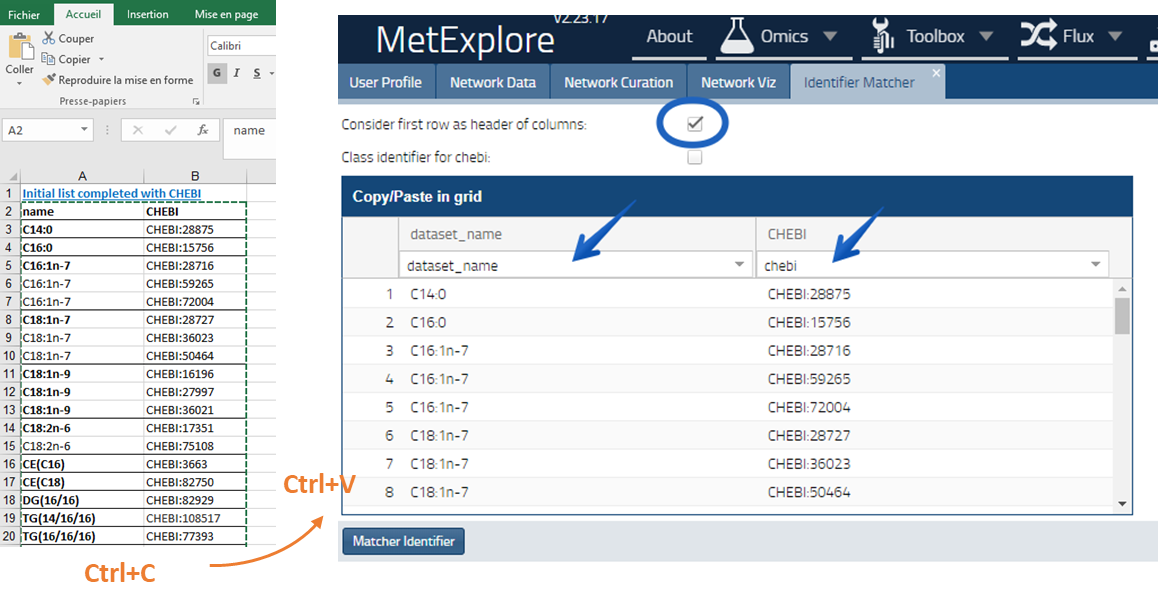


1. *Use the “Metabolite Identifier Matcher” tool in MetExplore*

**The “metabolite identifier matcher” tool allows you to perform 2 types of matching: a standard “exact” matching and a “class” matching**.

- The **“exact” matching** will try to match exactly the identifiers that you provide with the ones informed in the BioSource.

For instance, for the cholesterol ester C16 (CE(C16) – CHEBI:3663 – LMST01020005), it will search for network metabolites having one of these exact identifiers specified and will return “M_HC02020_l” and “M_HC02020_r”. You can check that these metabolites are indeed associated with the ChEBI:3663 by searching them in the “Metabolites” grid and look at their detailed information card.


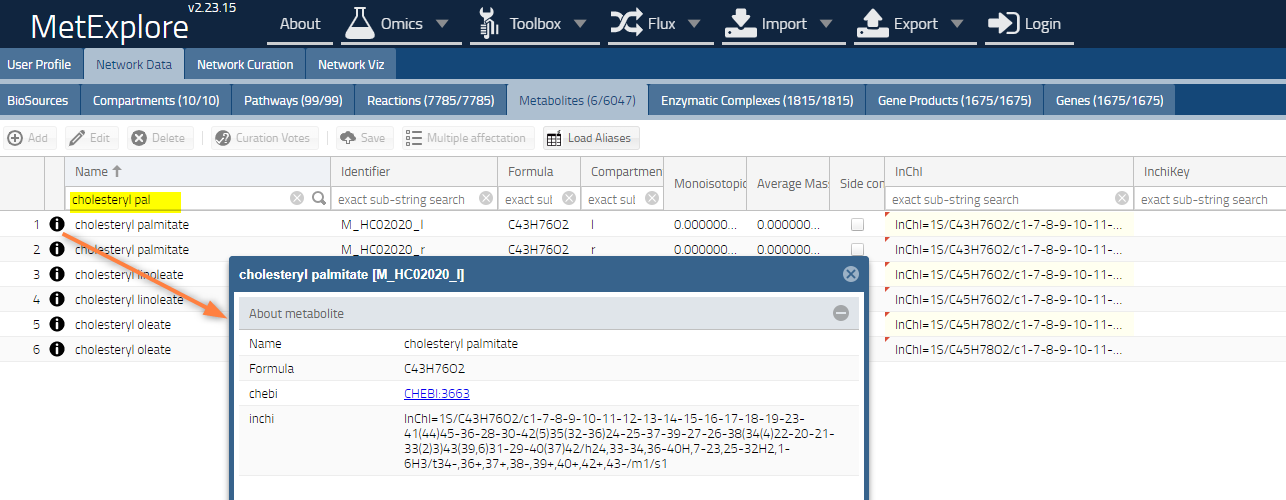


1. *Exact matching in MetExplore: example of the CE(C16) metabolite*

- The **“class” matching** will try to find more generic compounds corresponding to the sub-class, class, family … of the metabolite provided as input in case no exact match is found.

Indeed, in the metabolic networks, for some groups of molecules, each specific molecular species is not detailed but only the global family or class is present.

For example, in Recon2.2, all specific triglycerides (TG(16:0/16:0/16:0), CHEBI:77393; TG(18:0/16:0/16:0), CHEBI: 89751; …) are not detailed but are instead regrouped under the generic triglyceride class (“tag_hs”, CHEBI:17855).

By checking the **“class identifier for chebi and lipidmaps**” box, it is possible to **find the more generic “class” metabolites corresponding to specific data metabolites, if these specific metabolites are not detailed in the network (and their generic class is present)**. This is done based on the ChEBI and Lipidmaps identifiers, using the ontology defined in these databases. The distance from the dataset metabolites to the matched network metabolite is reported in the result table.

For example, the specific triglyceride TG(16:0/16:0/16:0) (CHEBI:77393) from the dataset will match with the global triglyceride "M_tag_hs" (CHEBI:17855) in the network, with a distance of 1.


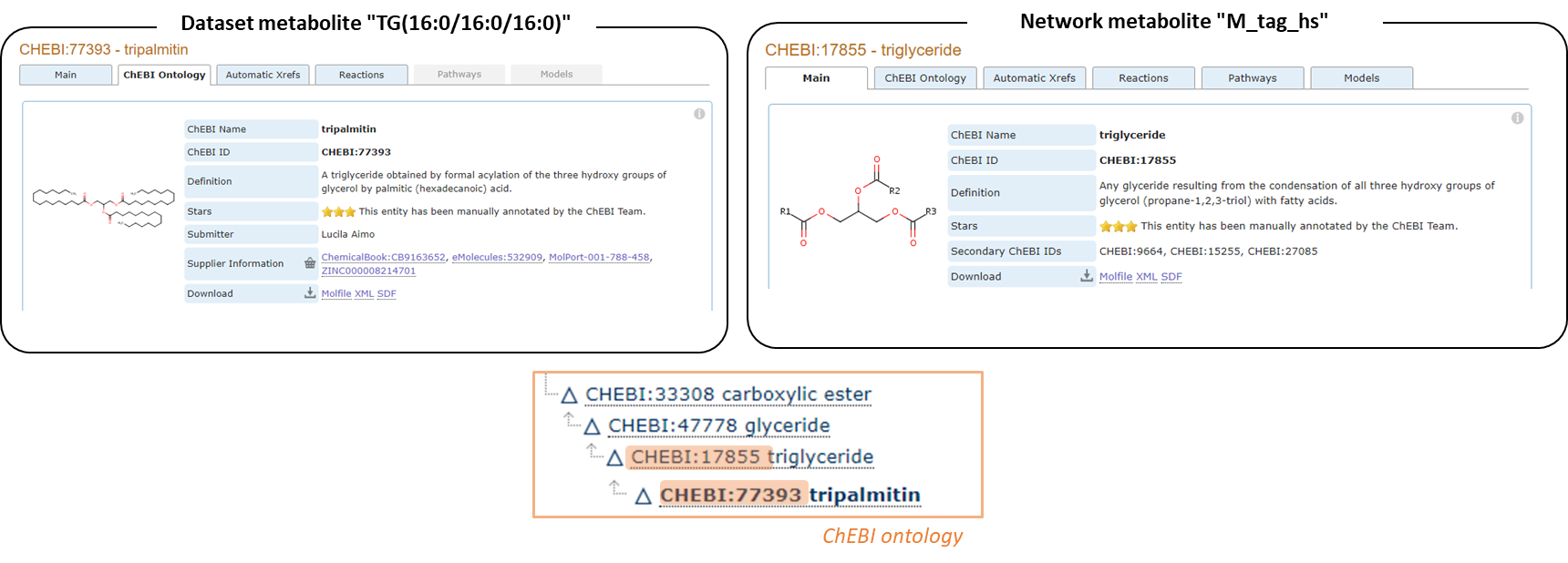


1. *Class matching in MetExplore: example of the TG(16:0/16:0/16:0) metabolite*

The specific triglyceride TG(16:0/16:0/18:0) (CHEBI:89751) from the dataset will also match with the global triglyceride "M_tag_hs" (CHEBI:17855) in the network, but with a distance of 2.


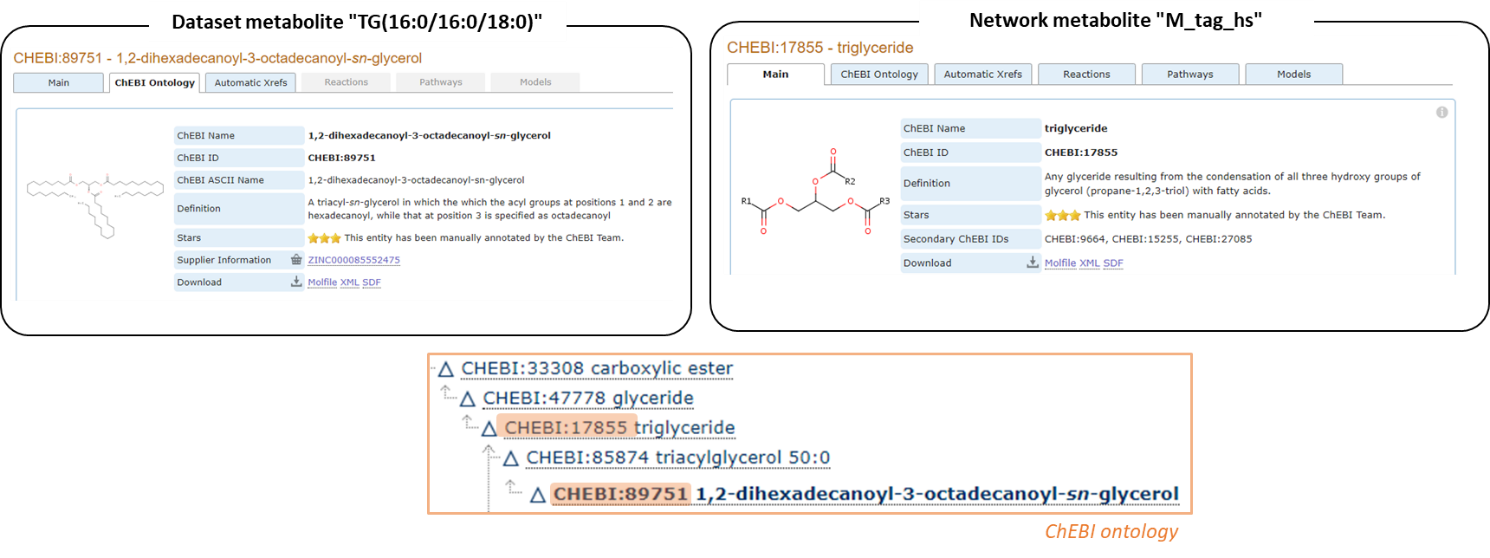


1. *Class matching in MetExplore: example of the TG(16:0/16:0/18:0) metabolite*

There is also the specific case, where **the anionic form (base) of the fatty acid is present in the network whereas the acid form in present in the dataset**. In this case, the base or acid form can be retrieved when using the “class” matching option. It will result in a matching distance of “0.1”.

For instance, the myristic acid (C14:0 in the dataset) is only present as “myristate” in the metabolic network.


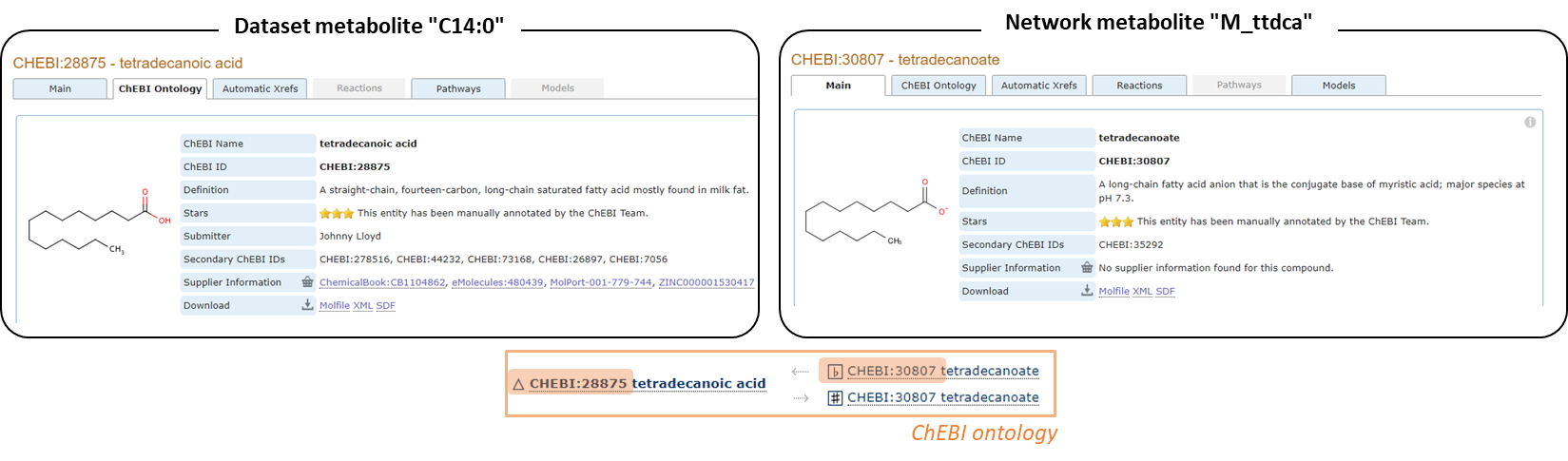


1. *Class matching in MetExplore: example of the C14:0 metabolite*

- *First perform an exact mapping of the NASH lipid dataset.*
- *Then perform a “class” matching of the NASH lipid dataset and compare the results.*


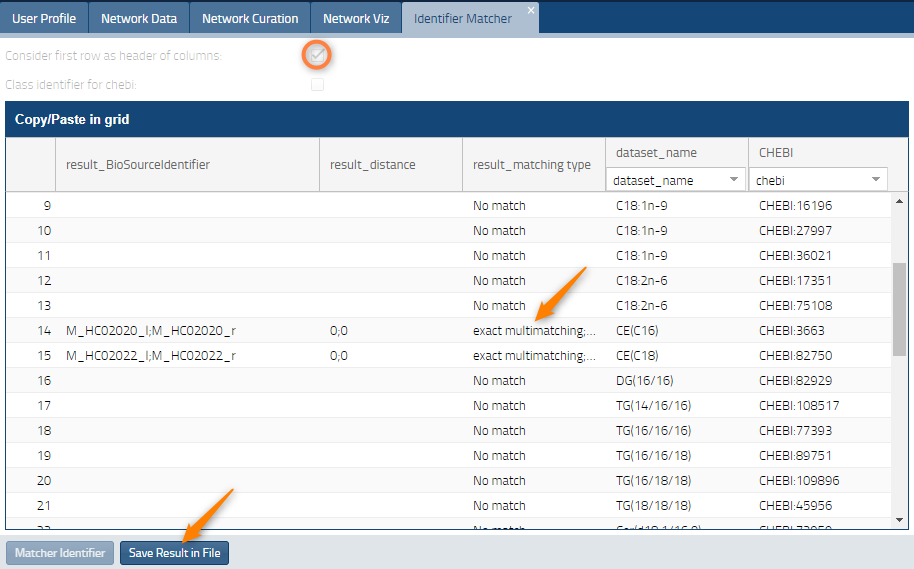


1. *Results of the exact matching from the NASH dataset in MetExplore*


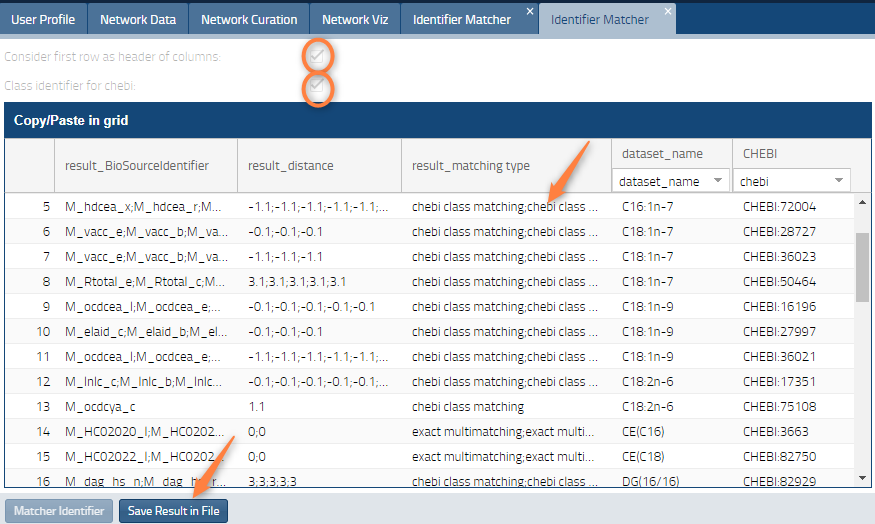


1. *Results of the class matching from the NASH dataset in MetExplore*

Results can be exported as an xlsx file by clicking on the “Save Results in File” button.

**The xlsx file contains 2 sheets:**

- The **“Dataset” sheet**, which contains the same columns as displayed in the MetExplore interface: the initial dataset list of metabolites (“dataset_name” and “dataset_chebi”) with 3 additional columns:
  - “**result_BioSourceIdentifier**”: contains the corresponding identifiers in the metabolite network.
  - **“result_distance**”: corresponds to the matching distance. “.1” corresponds to metabolites that are matched with the acid/base form.
  - “**result_mapping_type**”: indicates if the matching is exact or not.

If several identifiers match the dataset metabolite, these identifiers (as well as the corresponding “distance” and “matching_type” are separated by “;”.

2 complementary columns are also added in the case of a “class” matching:

- **“result_nbMapped”:** indicates the number of network metabolites that match with each dataset metabolite.
- **“result_averageDistance”:** is the average distance of all network metabolites that match with each dataset metabolite.
- The “**Metabolites**” sheet contains all the network metabolites with their name and identifier in the 2 first columns (“BioSource_name” & “BioSource_Identifier”) and 6 additional columns:
  - “**dataset_name**” contains the names of the data metabolites names that match with the network metabolites. If they are multiple data metabolites matching on the same network metabolite, they are separated by semi-colon;
  - “**distance**” indicates the matching distance for each metabolite in the “dataset_name” column, with the same order and also separated by semi-colon in case of multiple matches;
  - “**nbdataset_matched**” indicates the number of dataset metabolites that match with the network metabolite (corresponds to the number of metabolites listed in the “dataset_name” column;
  - **“average_distance**” is the mean of the matching distances of all dataset metabolites that match with the network metabolites (i.e., mean of the distances listed in the “distance” column).

Note that in case one dataset metabolite matches several times on the same network metabolite (because different identifiers are provided as input for the same metabolite) only the match with the minimal distance is kept in the “Metabolites” table (see example of “M_ps_hs” below).

**Potential cases with multiple matches:**

- Case #1 = **one dataset metabolite matches with multiple network metabolites**. This often happens when **several different entries** (different ChEBI identifiers) are provided as input. In this case, all matches are displayed in the output tables → see C18:1(n-9) example.
- Case #2 = **one dataset metabolite matches several times with the same network metabolite, but with different matching distances**. This also happens when several different entries (different ChEBI identifiers) are provided as input. In this case, all the matches are displayed in the "Dataset" output table, but only the minimal matching distance is kept and displayed in the "Metabolites" output table → see PS(18:0/23:0) example.
- Case #3 = **several distinct dataset metabolites match with the same network metabolite** → see "M_ps_hs" example.

**"C18:1(n-9)" example:**

Several ChEBI identifiers can be retrieved for the unsaturated fatty acid C18:1n-9, corresponding to the Z, the E or the non-specified isomer forms, as the analytical method does not allow to distinguish them. Therefore, depending on the CHEBI identifier that is considered, C18:1n-9 matches with:

- “M_ocdcea” (9Z-octadecenoate - CHEBI:30823) from CHEBI:16196 (9Z-octadecenoic acid, which is a conjugated base of CHEBI:30823) with a distance of 0.1;
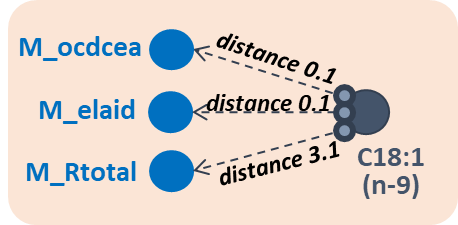

- “M_elaid” (9E-octadecenoate - CHEBI:30825) from CHEBI:27997 (9E-octadecenoic acid, which is a conjugated base of CHEBI:30825) with a distance of 0.1;
- "M_Rtotal” (fatty acid anion – CHEBI:28868) from CHEBI:36021 (octadec-9-enoic acid, which is a conjugated base of CHEBI:132944) with a distance of 3.1.

→ All 3 matches are displayed in the result tables:


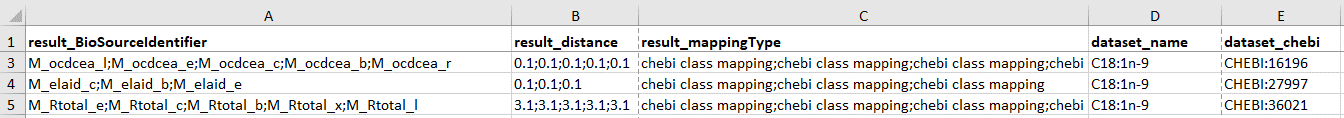


1. *“Dataset” output table for matching of C18:1n-9 in MetExplore*


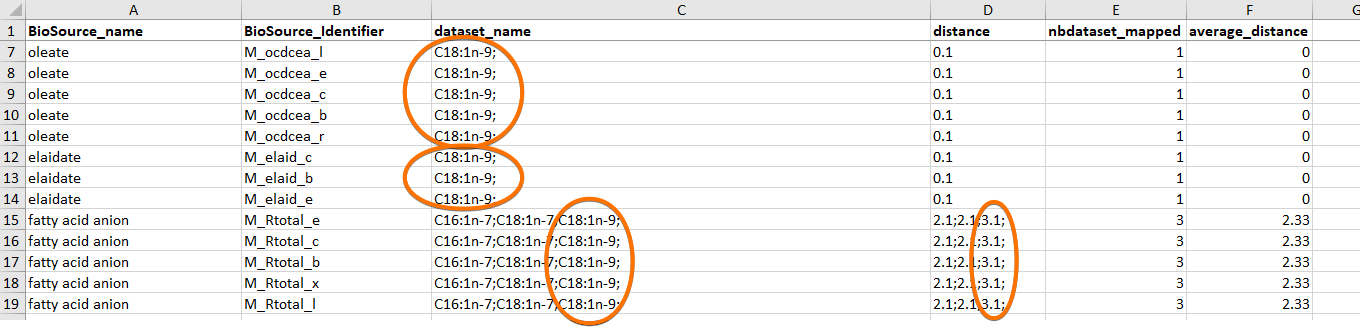


1. *“Metabolites” output table for matching of C18:1n-9 in MetExplore*

**"PS(18:0/23:1)" example:**

Several ChEBI identifiers can be retrieved for the phosphatidyl-serine, depending whether the isomery of the C23:1 fatty acid is specified (1-stearoyl-2-(8Z,11Z,14Z-icosatrienoyl)-sn-glycero-3-phosphoserine; CHEBI:84512) or not (phosphatidylserine(18:0/20:3); CHEBI:90435).
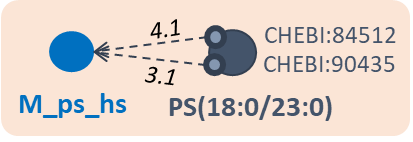


Depending on the CHEBI identifier that is considered, PS(18:0/23:1) matches with "M_ps_hs" with a matching distance of 4.1 or 3.1.

→ in the "Dataset" table: the 2 matches are displayed


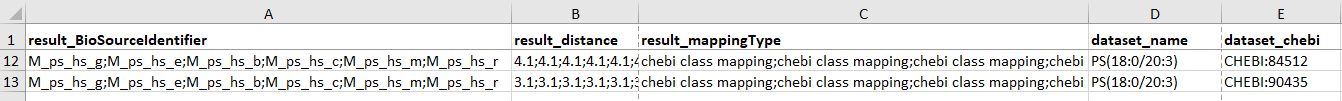


1. *“Dataset” output table for matching of PS(18:0/23/1) in MetExplore*

→ in the "Metabolites" table: only the minimal distance is displayed.


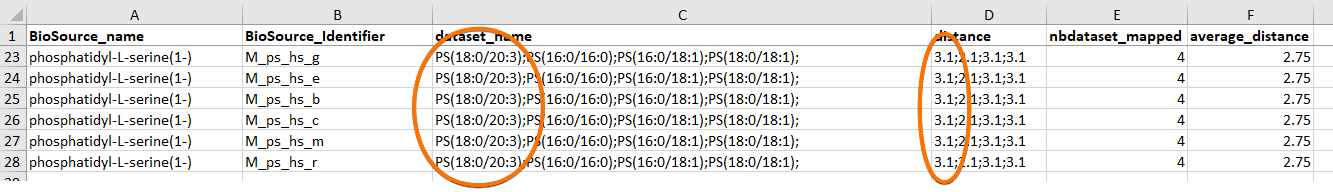


1. *“Metabolites” output table for matching of PS(18:0/23/1) in MetExplore*

**"M_ps_hs" example:**

Several distinct dataset metabolites match on the "M_ps_hs" network metabolite: PS(18:0/23:1), PS(16:0/16:0), PS(16:0/18:1) and PS(18:0/18:1). Note that PS(18:0/23:1) and PS(18:0/18:1) match each twice with "M_ps_hs" from 2 distinct ChEBI entries (see previous example).


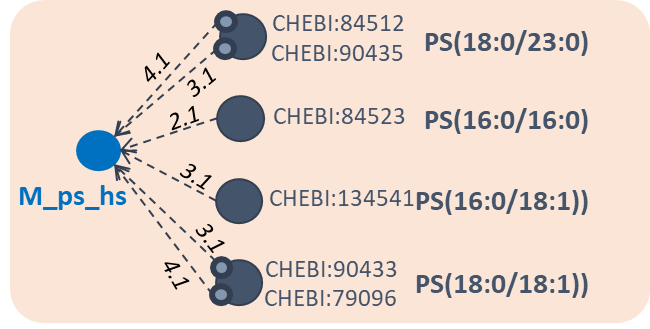

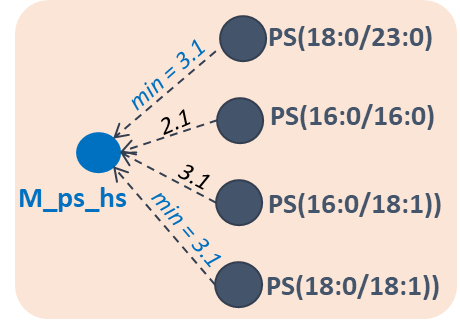


→ in the "Dataset" table: all the matches are displayed

→ in the "Metabolites" table: only 4 matches are displayed, one per each distinct dataset metabolites (for PS(18:0/23:1) and PS(18:0/18:1), only the minimal matching distance is kept).


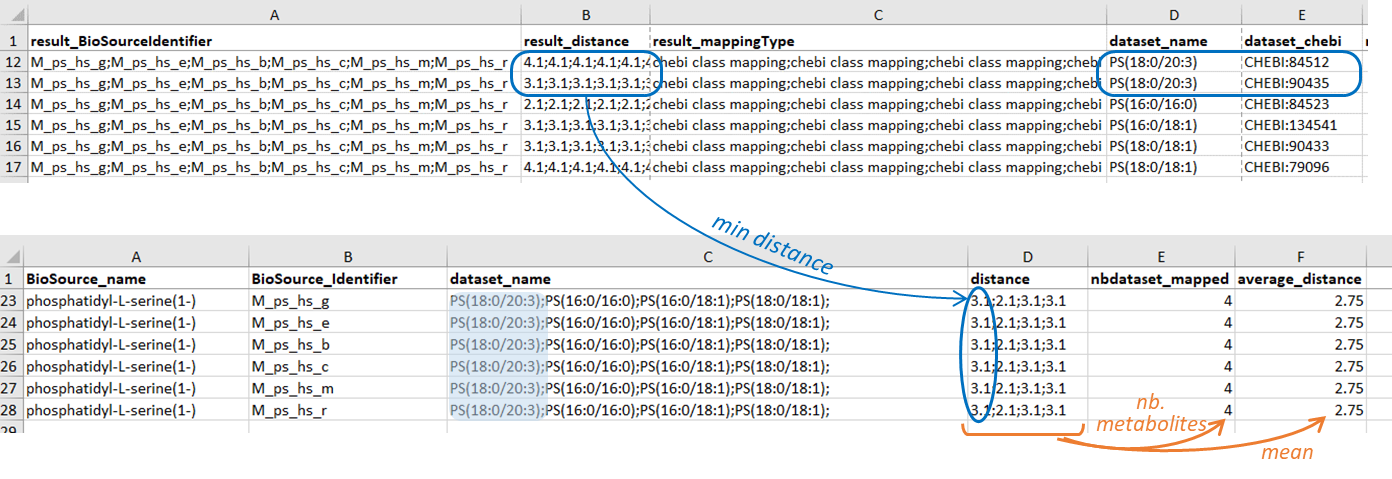


1. *“Dataset” & “Metabolites” output tables for matching of M_ps_hs in MetExplore*

# Mapping obtained metabolite identifiers

- **Getting the list of network metabolites to map**

**To map your dataset metabolites, you need to import in MetExplore the list of corresponding identifiers specific to the BioSource you are working on.** This list can be extracted from the "Metabolites" table obtained using “metabolite identifier matcher” (as we did in the previous section).

To extract this list of network identifiers corresponding to the NASH dataset metabolites:

- *Go to the "Metabolites" sheet of the xlsx file obtained from the “metabolite identifier matcher”*
- *Filter in the table to display only the rows with matching dataset metabolites: select the header row of the table, and then click "Filter" in the excel "Data" tab.*
- *Drop-down arrows will appear in the header of each column.*
- *Click the drop-down arrow of the "dataset_name" column → the Filter menu appears.*
- *Uncheck the box next to "empty" → the table is filtered to display only the rows that have a non-empty "dataset_name" cell. You should get a list of 84 metabolites.*
- *Copy / paste the displayed list in another excel file or tab.*

**Note that the list of BioSource identifiers obtained using the “metabolite identifier matcher” often needs to be reformatted for the purpose of the mapping, to deal with some ambiguous cases**. Indeed, you noticed that you have a higher number of mapped metabolites (84) than the initial number of metabolites in the NASH the signature. This is due to 2 main reasons:

- **First, since the metabolic network includes the different cellular compartments, input metabolites can be mapped on different compartments.** For example, the palmitic acid (C16:0, M_hdca) is found in the cytosol (M_hdca_c), the mitochondria (M_hdca_m), the peroxisome (M_hdca_x), the lysosome (M_hdca_l), the endoplasmic reticulum (M_hdca_r), the extracellular space (M_hdca_e) and a "boundary" compartment (M_hdca_b).
- **The "extracellular space" (e) and "boundary" (b) compartments** include metabolites that can be exchanged by the cell or organism with its environment (*e.g.,* metabolites that are produced or consumed by the cell to or from the cell culture medium). These compartments are added in the metabolic network to account for the exchange reactions with the environment, but **these metabolites are actually not measured when performing intracellular metabolomics analyses, and therefore should be removed from the list.**
- Other compartments correspond to **intracellular compartments** (Cytosol, mitochondria …). Because there are no information about the cellular localization of the identified metabolites in the data, **it is necessary to keep all the metabolites mapped in these intracellular compartments**.
- Second, as we have seen previously, a dataset metabolite may match with several BioSource metabolites. In the case that the matching distances are different, it might be more relevant to keep only the matched network metabolite that is the closest (i.e., with the shortest distance). If the matching distances are similar, both could be kept.

For example, in the NASH lipid dataset,

- The unsaturated fatty acid "C16:1n-7" matches both with “M_hdcea” (9Z-hexadecenoate), with a distance of 0, and with “M_Rtotal” (fatty acid anion), with a distance of 2. In this case, it seems more relevant to keep only the "M_hdcea" metabolite.
- similarly, “C18:1n-9” matches both with “M_ocdcea” (9Z-octadecenoate) and “M_elaid” (9E-octadecenoate) with a distance of 0, and with “M_Rtotal” (fatty acid anion), with a distance of 2. In this case, because both isomers Z and E are present in the network, both "M_ ocdcea" and "M_elaid" metabolites should be kept.
- Also, in our example, “M_Rtotal” might be removed from the list of mapped BioSource metabolites since none of the dataset metabolites solely matches with it.

A "cleaned" list of BioSource metabolite identifiers to be used for mapping is provided in the supplementary file 5 (sheet "FinalList_forMapping"). "_b" and "_e" metabolites, as well as "M_Rtotal" metabolites have been removed from the initial list.

**This final list contains 53 distinct network metabolites.**

**We will use the columns "BioSource_Identifier" and "nbdataset_mapped" (which corresponds to the number of metabolites from the initial NASH dataset that have been mapped on each network metabolite).**

- **Performing mapping in MetExplore**


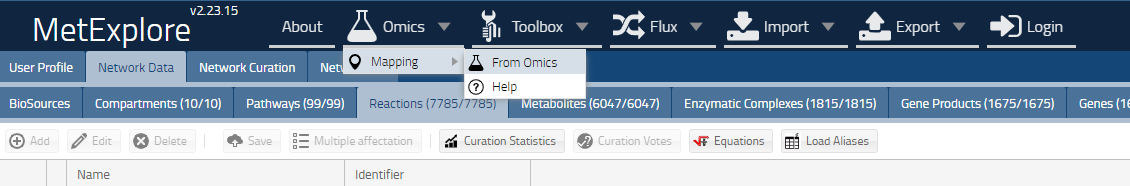


1. *Mapping data metabolites in MetExplore*

- *Select “Omics" → “Mapping” → "From Omics" in the menu at the top of the page: this will open a “Mapping” window*
- *Check the “Consider first row as header of columns” box if you have copied the first line containing the columns headers.*
- *Enter a “Mapping name”*
- *Copy the data from the* [*Excel file*](https://docs.google.com/spreadsheets/d/1DhIw3A_ylYS3ex7QCfOEi1sbVeL77FKyPAgtNIoX3kk/edit#gid=1318852603) *(select only the columns "BioSource_Identifier" and "nbdataset_mapped") and paste it directly in the mapping grid, using Ctrl+V.*

Note that the first column of your input data must be the type of identifiers that will be used to map (*i.e.,* find) the corresponding elements (in our case the metabolites) in the biological network. Following columns are facultative and correspond to numeric values in different conditions. **In our case, the condition is the number of matched metabolites from the initial NASHdataset.**


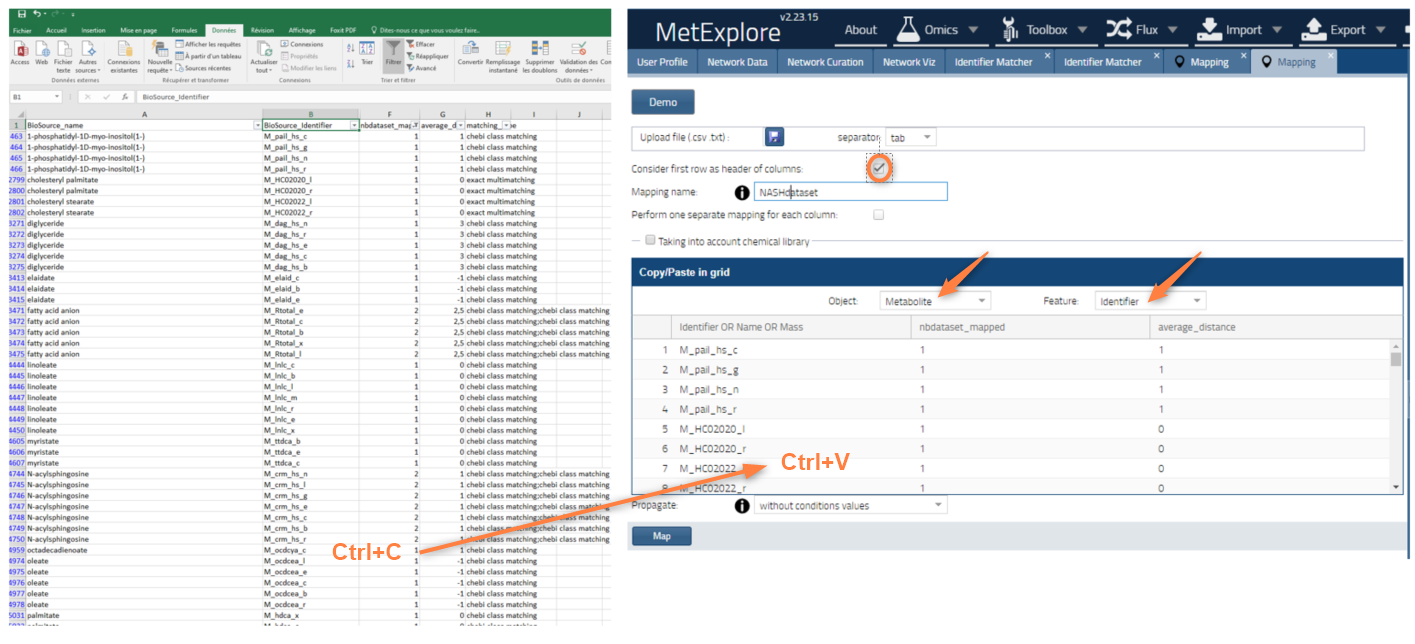


1. *Importing data metabolites for mapping in MetExplore*

Mapping can be performed for all biological objects stored in a metabolic network (pathways, reactions, metabolites, enzymes, gene products and genes).

- *Select “Metabolite” as the type of biological objects you want to map in the "Object" menu*
- *Select “Identifier” as feature, to indicate that you are providing BioSource identifiers.*

Note that the mapping could also be made from the name.

- *Click on the “Map” button*

Once the mapping has been achieved, a new column “Identified” is displayed, indicating, for each input metabolite, whether it has been found in the network (true) or not (“false”).

Some statistics are also displayed:

- “Nb. Data”: the initial number of metabolites in the input dataset;
- “Nb mapped”: the number of input metabolites that have been successfully mapped in the network;
- “Nb. Data in the network”: the number of corresponding network metabolites;

Note that these two last numbers might differ if two input metabolites map on the same network metabolite for instance.


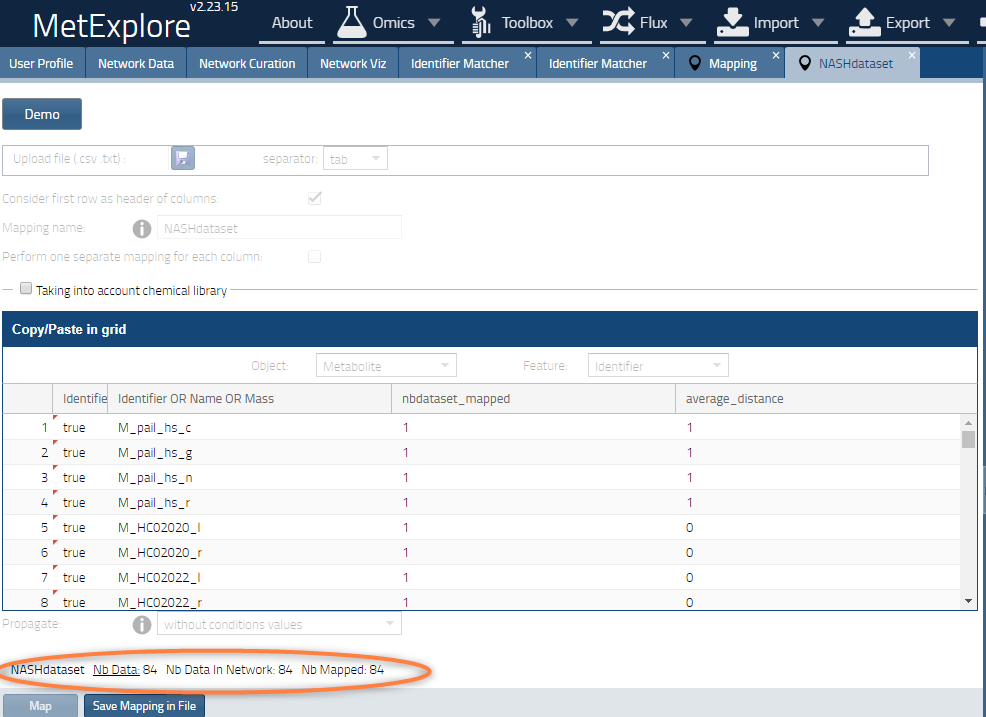


1. *Results of the NASH dataset mapping in MetExplore*

The mapping can be exported using the "Save Mapping in File" button in the mapping window: it will be exported as a json file and can be later imported back in MetExplore using the "Import" menu in the top bar, and selecting "Import Mapping from file".

# Identifying pathways and reactions related to the mapped data

Results of the mapping are also displayed in the “pathways”, “reactions”, and “metabolites” grids of the network data, where a new column entitled with the mapping name is added.

- In the **“Metabolites” grid**, this main column is subdivided into several sub-columns: the first one ("identified") indicates if the metabolite was in the dataset, and the next ones correspond to the values ​​of the different conditions (in our case, the number of matched metabolites from the initial NASH dataset).
- *Sort the "identified" column to display the mapped metabolites at the top of the grid.*


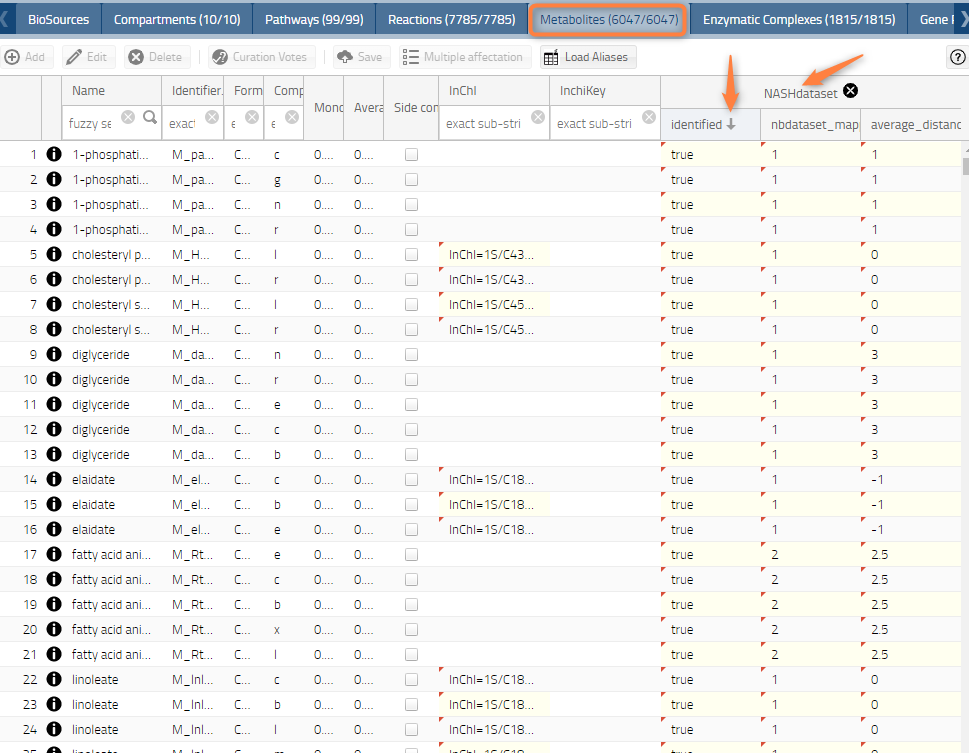


1. *Results of the metabolite mapping displayed on the metabolites grid in MetExplore*

- In the **“Reactions” grid**, the added column for mapping results indicates, for each reaction, the number of metabolites that are present in the NASH dataset.

For instance, the “Sphingomyelin synthase” reaction uses 4 metabolites that are present in the dataset: “M_pchol_hs”, “M_crm_hs”, “M_sphmyln” and “M_dag_hs” among the 5 metabolites that are either consumed or produced by this reaction, leading to a coverage of 80% by the data. Similarly, 2 dataset metabolites are mapped on the elaidic acid exchange reaction out of the 2 metabolites used by this reaction (coverage = 100%).


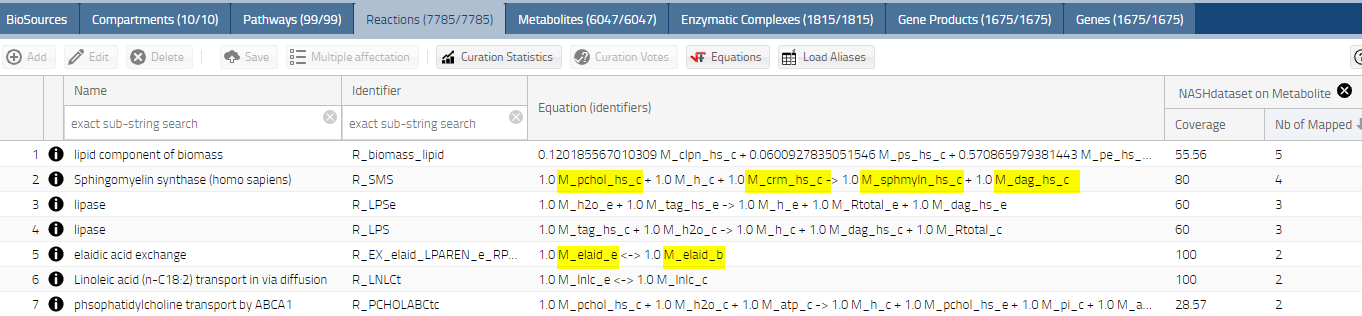


1. *Results of the metabolite mapping displayed on the reactions grid in MetExplore*

- Similarly, in **the “Pathways” grid**, the added column indicates the number of data metabolites that are found in each pathway (“Nb of mapped”) and the proportion of the pathway that these mapped metabolites represent (“coverage”). **The over-representation of the mapped metabolites in each pathway is tested using a Right tailed Fisher Exact Test (“p-value” column). The p-values are corrected to account for the multiple tests performed for all pathways.** Bonferroni and Benjamini-Hochberg corrected p-values are presented in the grid. *** indicates a p-value < 0.0001; **indicates a p-value < 0.001; *indicates a p-value < 0.05.

Note that the results of this pathway over-representation analysis gives you an idea about the global localization of your dataset metabolites. However, you need to be careful when interpreting these results in terms of statistical representativeness as they might be biased by the type of analyses that the data are issued from. Indeed, not all metabolites can been identified by the metabolomics analyses (and *a fortiori* by the lipidomics analyses) so that some pathways may not be covered at all by your analyses whereas others will be artificially over-covered.


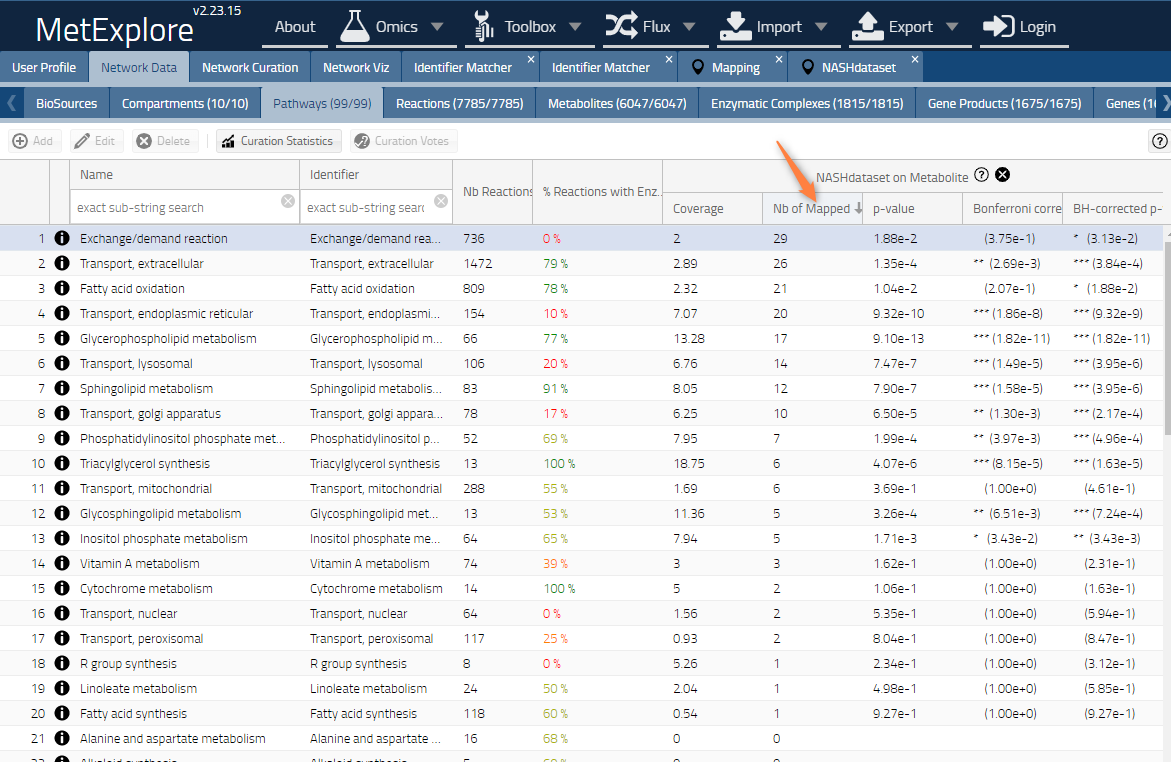


1. *Results of the metabolite mapping displayed on the pathways grid in MetExplore*
2. **Visualizing mapped data and subnetworks of interest**

**Objective:** In this section, we will show **(1) how to create a network which is the union of 3 metabolic pathways of interest** (e.g., the “Glycerophospholipid metabolism” pathway, the “Sphingolipid metabolism” pathway and the “Phosphatidylinositol phosphate metabolism” pathway), **(2) how to visualize it** and **(3) how to highlight mapped metabolites**. This requires to first put all the reactions belonging to at least one of the pathways of interest in the cart and then move to the visualizing tool to view reactions and mapped metabolites.

### The “Cart” concept: to interactively create and visualize networks based on selection of reactions (e.g. the union of all reactions belonging to specific pathways or all reactions taking place in the mitochondria), MetExplore use the concept of “Cart”, as any online shopping system. The Cart content is displayed in the right panel of MetExplore.

# Creating a subnetwork of interest based on pathways

In this first sub-section, we will learn how to select a group of reactions belonging to pathways of interest, and put them in the "cart" for visualization.

Following the example of NASH lipidomic signature, we would ideally like to create a subnetwork including all the pathways that contain at least one mapped lipid from the dataset.

These pathways can be identified in the “Pathways” grid by selecting all the pathways with a “nb of mapped” value higher than 0.

- *Sort the “Nb of mapped” column in descending order by clicking twice on the header of the column or by clicking on the arrow on the right hand corner of the column header.*

**→ 17 metabolic pathways contain at least one mapped lipid from the NASH dataset (3492 reactions).**


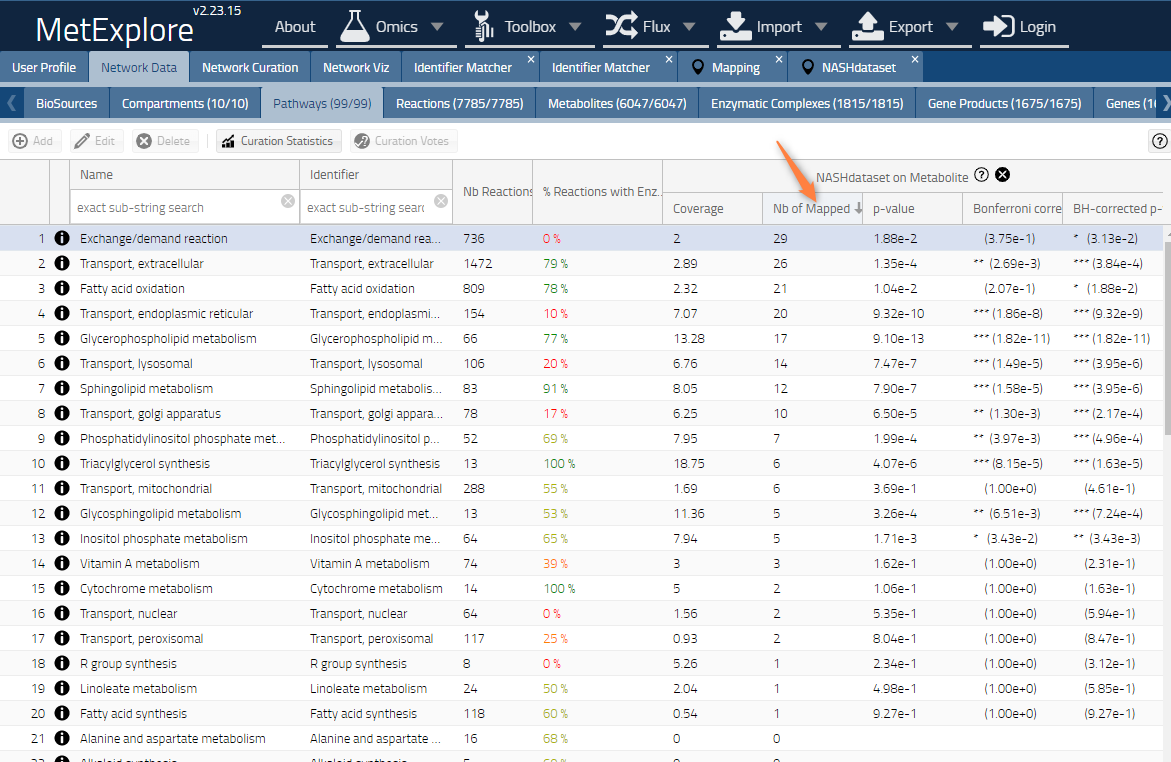


1. *Sorting the pathways according to the number of mapped metabolites*

In this practical, because visualization of large networks (more than 1000 reactions) might be relatively slow, we will focus only on a few of these pathways that have the lowest p-values and that are not transport pathways: **“Glycerophospholipid metabolism”, “Sphingolipid metabolism” and “Phosphatidylinositol phosphate metabolism”.**

- *Select these 3 pathways (hold CTRL for multiple selection)*
- *Right click and filter these pathways ( → "new filter on selection"):*

**All the grids are now filtered to display only the elements belonging to these pathways.**

This can be observed in the numbers displayed at the top of each grid. The first number is the number of elements corresponding to the filter (e.g., 201 reactions are present in the 3 selected pathways). The second number is the total number of the given element in the network (e.g. 7785 reactions).


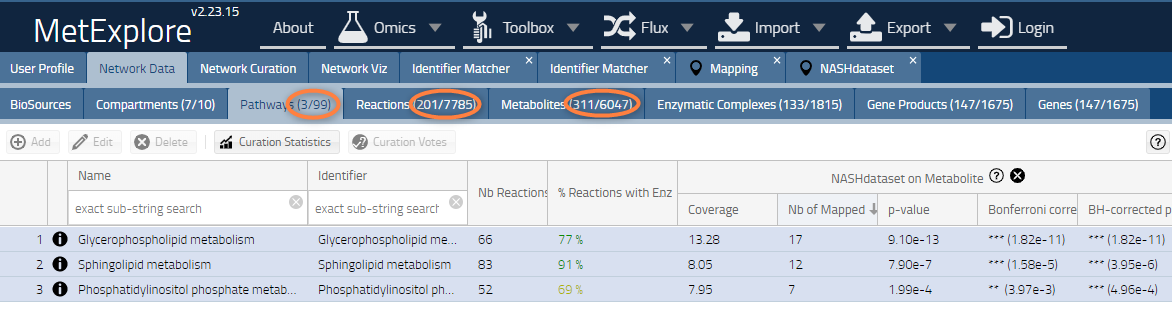


1. *Filter on selected patways in MetExplore*

We will visualize all the 201 reactions belonging to these 3 pathways. To do so:

- *Go to the "reactions" grid*
- *Right click on the reactions list and select "copy all to cart"*


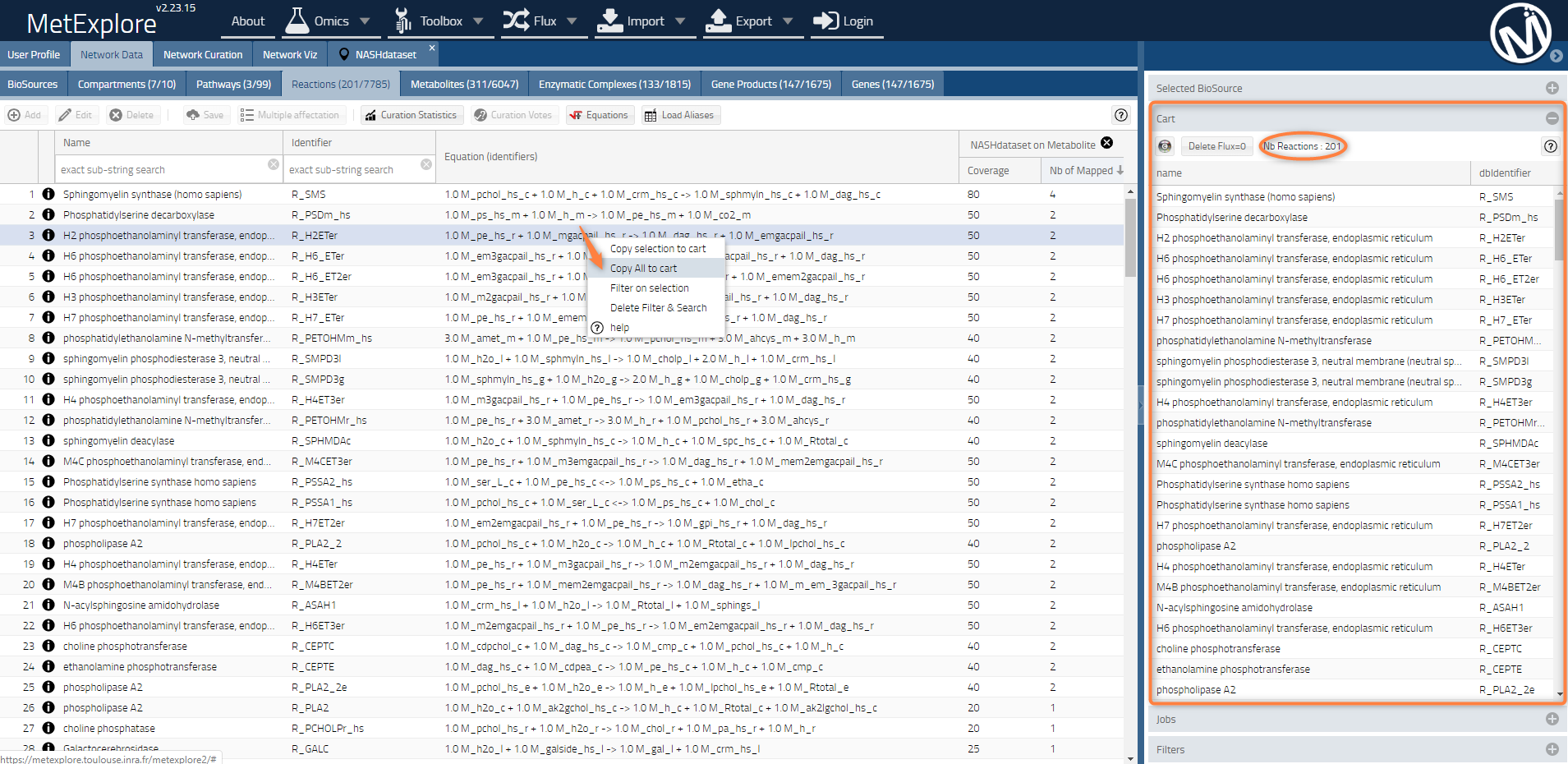


1. *Copying all reactions filtered from pathways in the MetExplore cart*

Note that you can filter out only some specific reactions by selecting them and choosing "copy selection to cart".

All the 201 reactions belonging to the “Glycerophospholipid metabolism”, “Sphingolipid metabolism” and “Phosphatidylinositol phosphate metabolism” pathways are now displayed in the "Cart", in the right panel.

# Visualizing the selected subnetwork

The aim of this second sub-section is to display the sub-network formed by all the selected reactions.

Complete documentation on the usage of the visualization can be found here: <https://metexplore.toulouse.inra.fr/metexploreViz/doc/documentation.php>

- **Global visualization**

**Visualization can be achieved using the “Network viz” panel**.

In MetExplore, visualization of a network or sub-network can be achieved either by importing a json file (e.g., saved network visualization from a previous MetExplore session) or from a list of reactions selected in the biosource grid and put into the cart.

**In this section, we will perform the visualization based on the reactions present in the cart.**

- *Go to the “Network viz” panel and click on the “MetExplore Selection” button.*


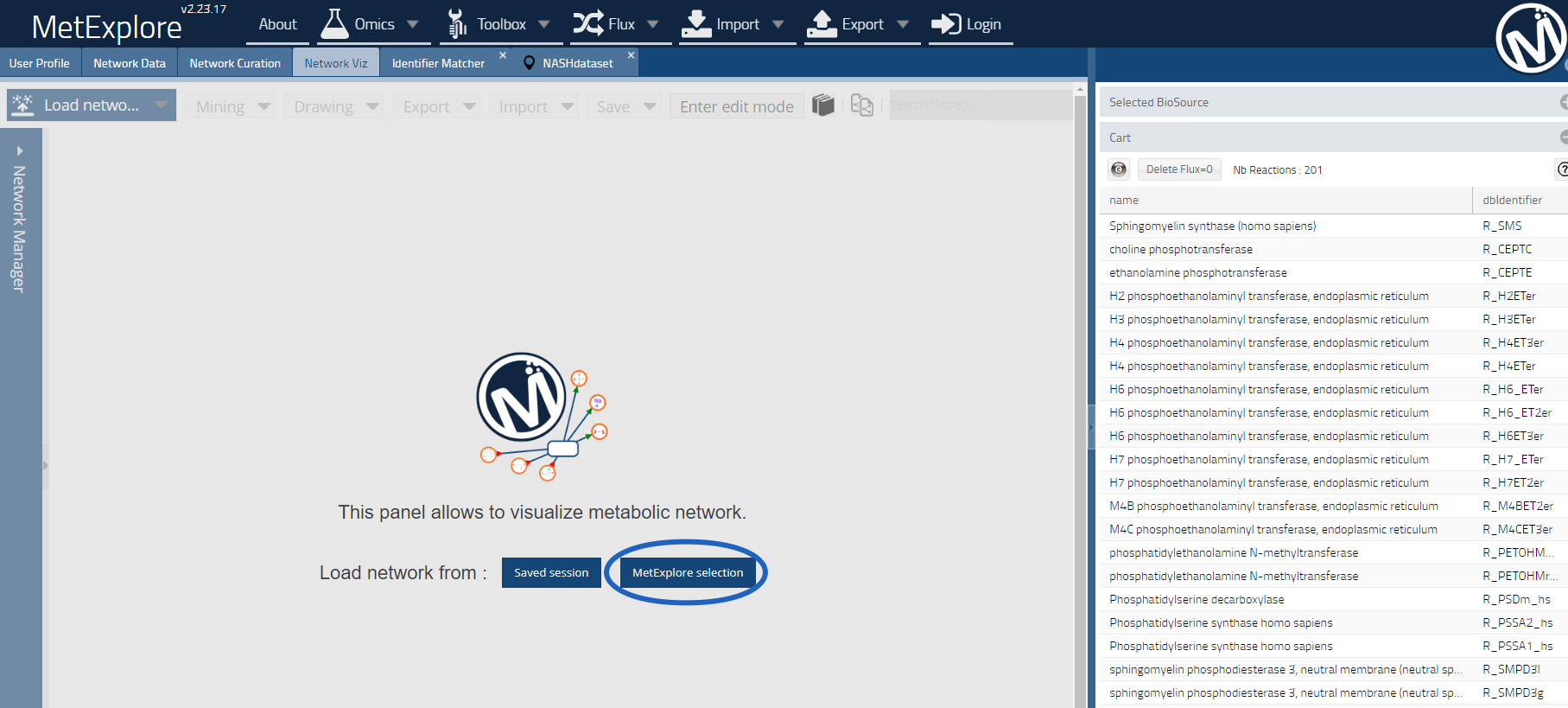


1. *Network visualization window in MetExplore*

The resulting network is drawn in MetExplore.


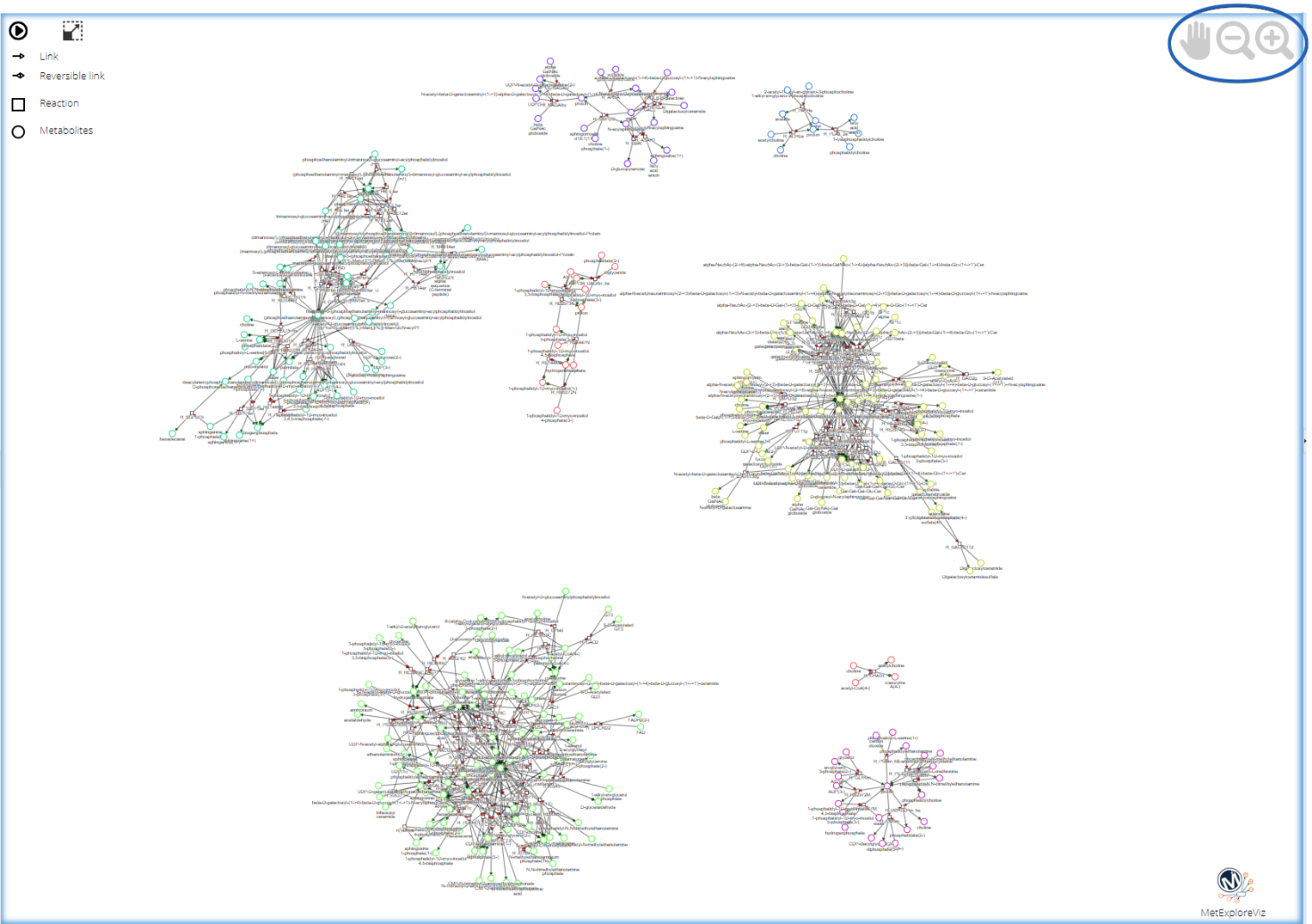


1. *Visualization of the 3 selected metabolic pathways in MetExplore*

- *Click on the lecture/pause button to pause or restart the animation.*

You can move the drawing or zoom in or out using either the mouse wheel or using the 3 buttons at the top right corner.

**In the visualization, reactions and metabolites are displayed as nodes: reactions with rectangles and metabolites with circles.** The edit mode allows modifying the form, the size and the labels of the nodes. These features will not be developed in this tutorial, but detailed information can be found in the MetExplore Viz documentation.

- **Highlighting cellular compartments and metabolic pathways**

Metabolites (circles in the representation) have different colors. This color correspond to the cellular localization of the metabolite (cytoplasm, mitochondria …). **You can also highlight the compartments by drawing convex hull around them:**

- *Expand the "Compartments" sub-panel on the left hand side, in the "Network Manager" panel.*
- *Click on "Highlight compartments".*

Or

- *Click on "Drawing" → "Highlight component" → "Compartments"*


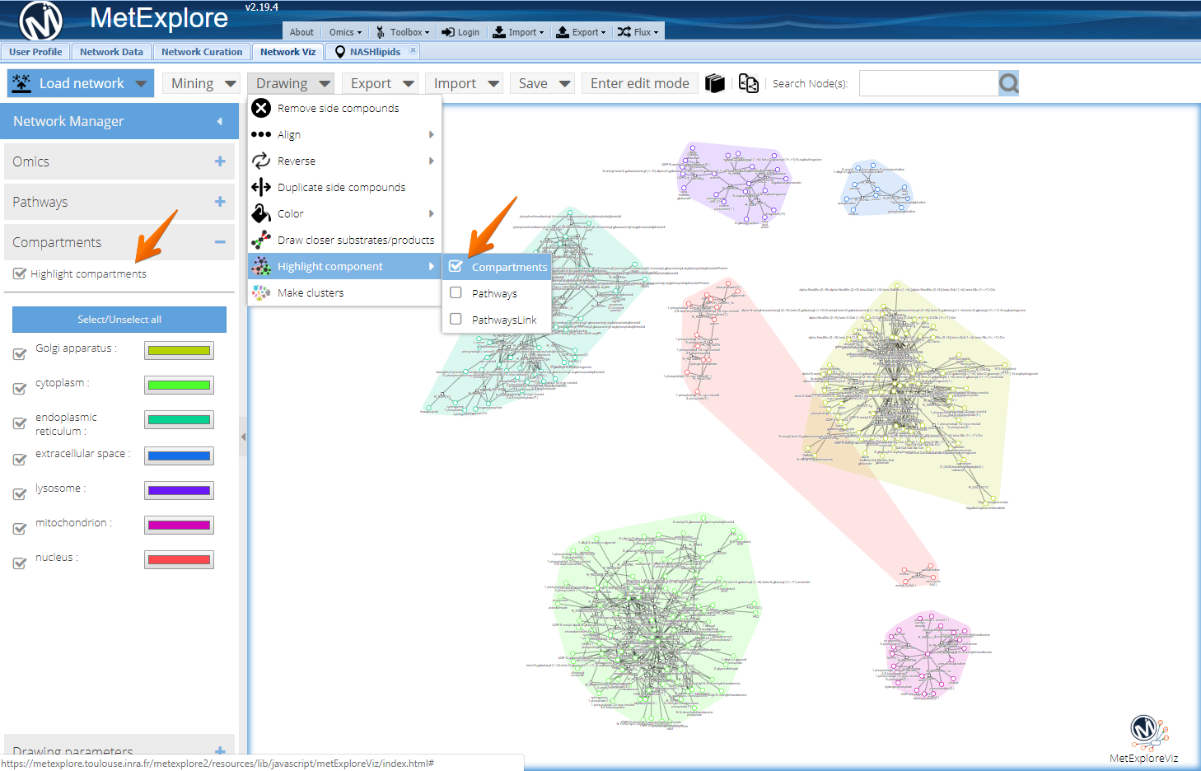


1. *Highlighting cellular compartments in the MetExplore visualization*

Note that since we did not include the transport reactions pathways in our selection the different cellular compartments appear "disconnected".

You can similarly **highlight the metabolic pathways with hulls or different colors on the reaction links**.

- *Expand the "Pathways" sub-panel on the left hand side, in the "Network Manager" panel.*
- *Click on "Highlight pathways" or "Highlight pathways on links".*

Or

- *Click on "Drawing" → "highlight component" → "Pathways" or "PathwaysLink"*

It is also possible to display only one pathway by selecting only this pathway in the list of pathways.


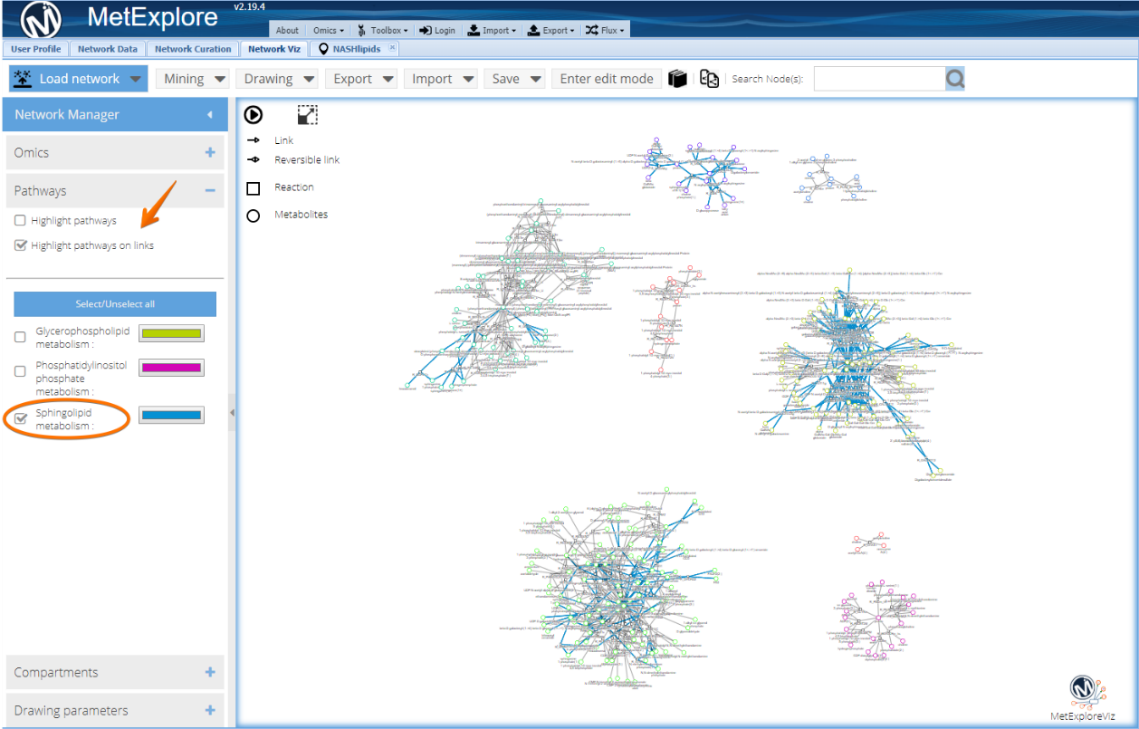


1. *Highlighting metabolic pathways on links in the MetExplore visualization*

- **Duplication of side compounds**

You notice that some nodes create hubs in the network, as they are connected to many others nodes. These nodes correspond to metabolites such as H_2_O, proton …, which are involved in many metabolic reactions. They are called **"side-compounds"**. To ease the visualization it is recommended to either remove or duplicate the side compounds. Note that considering that a metabolite is a side compound might sometime depend on the reaction, so that side compounds are not defined and removed *a priori*.

To duplicate or remove a side compound directly from the visualization window:

- *Select the metabolite you identified as being a side compound (e.g., proton)*
- *Right click and select "Duplicate nodes as side compounds" → "this node"*


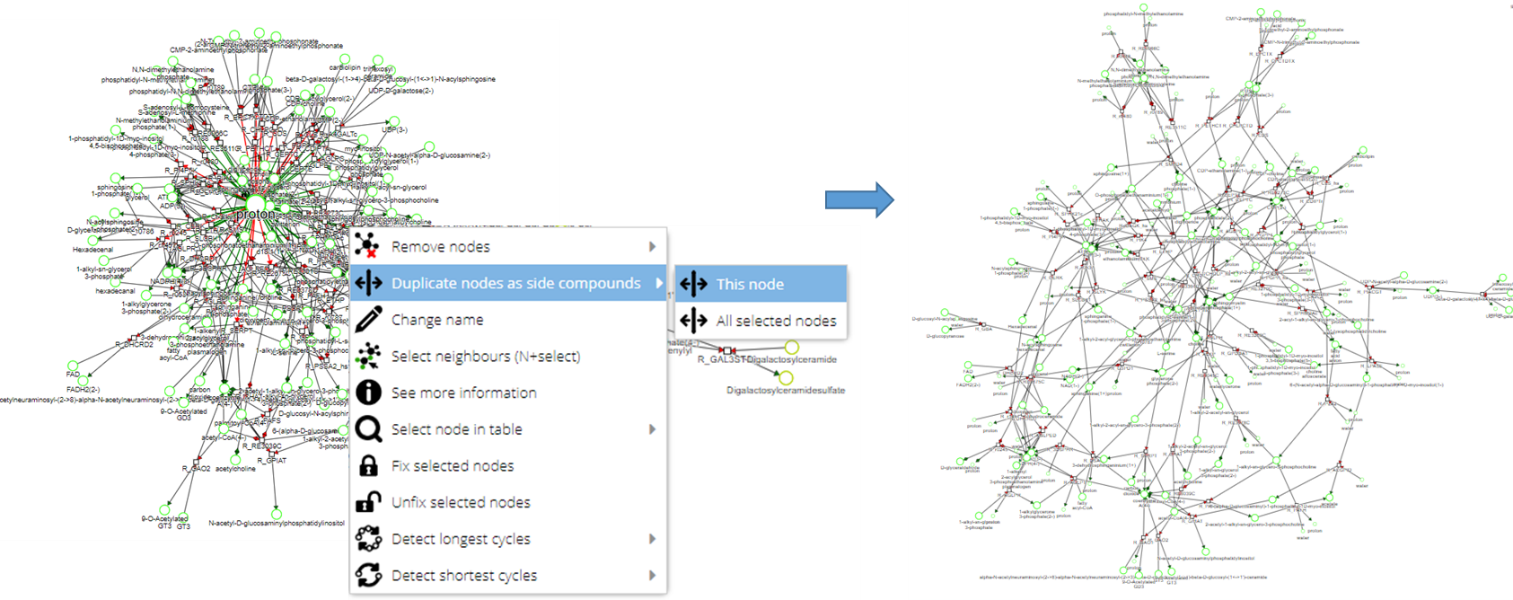


1. *Duplication of side-compounds metabolites in the MetExplore visualization*

You can reiterate this process for each metabolite that you consider as side compound.

A faster way is to specify metabolites as side compounds in the "metabolites" grid and then duplicate or remove them all at once.

- *In the "metabolites" grid, check the box in the "side compound" column for the metabolites you consider as side compounds.*
- *In the "network viz" tab, click on "Drawing" → "Duplicate side compounds"*


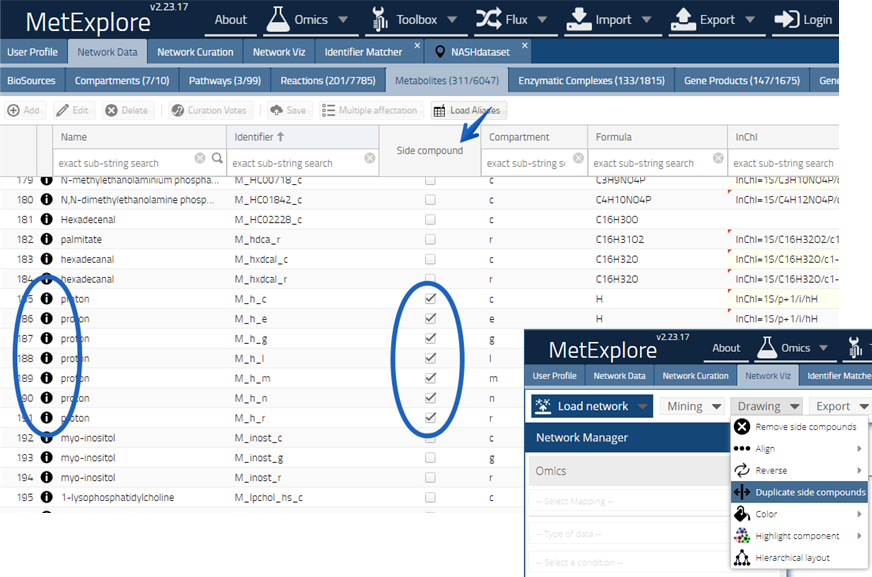


1. *Defining and duplicating side-compounds metabolites from the metabolites grid in MetExplore*

- *Define as side compounds and duplicate all the metabolites that are in the "SideCompounds" sheet of the xlsx file.*

You should get a much less dense network.


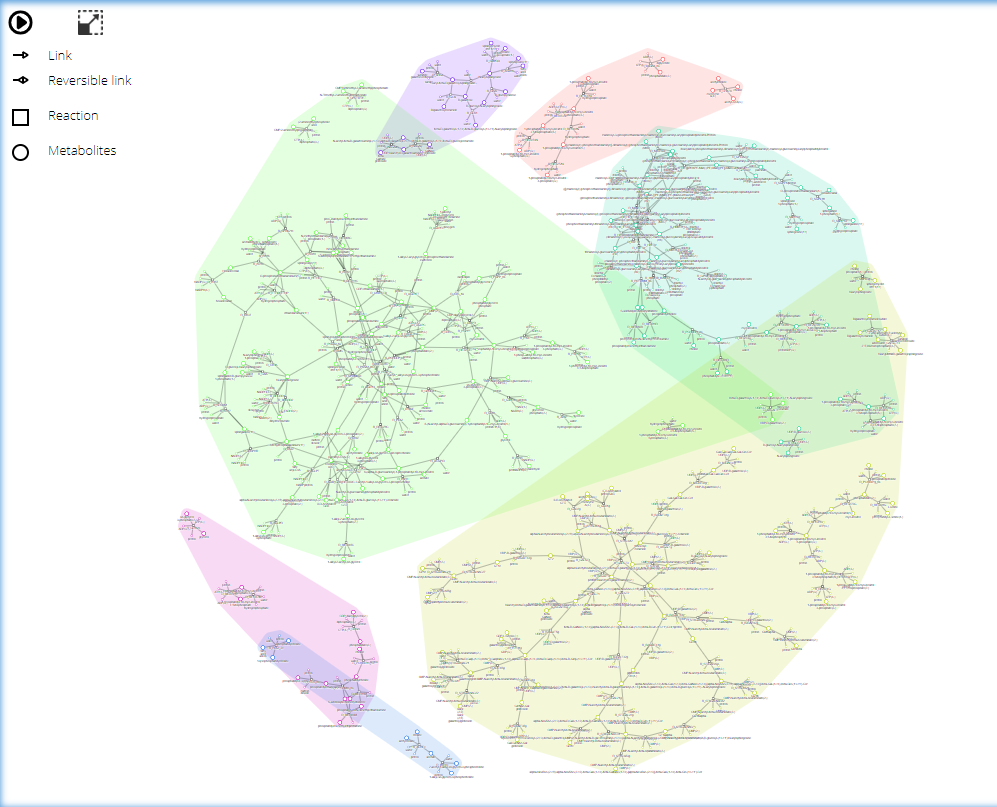


1. *MetExplore visualization of 3 selected metabolic pathways with duplicated side-compounds*

# Highlighting mapped data

**The aim of this last sub-section is to display the NASH dataset metabolites on the selected and visualized subnetwork**.

The mapping on the dataset metabolites has been done in the 2.3 section and can now directly be accessed in the "Network Viz" tab.

- *Expand the "****Omics****" menu in the left panel*
- *Select your dataset in the dropdown menu*

Mapped metabolites are surrounded in red in the visualization.

- *Select the* ***type of data*** *(if you have associated values with your metabolites)*

Colors will be assigned to nodes according to the numerical values and depending on the data type:

- for discrete values, a color is assigned to each value;
- for continuous values, a color gradient (by default, from yellow to blue) is created and each node is assigned a color accordingly, using a linear scaling.

In our case, values correspond to the number of the metabolites from the initial NASH signature that matched on each network metabolite, so that they are discrete values.

- *Select a* ***condition****: either "nb. of dataset metabolites mapped" or "average distance"*


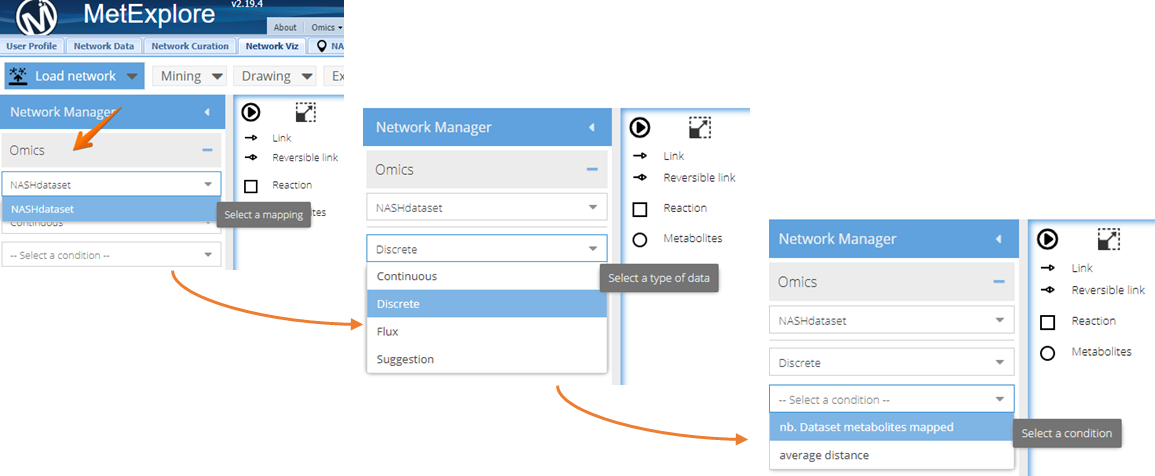


1. *Highlighting mapped metabolites in the visualized sub-network*

Mapped metabolites are now highlighted in the subnetwork (filled circles), with colors corresponding to the values of the chosen condition, in our example the number of NASH metabolites mapped on each network metabolite.


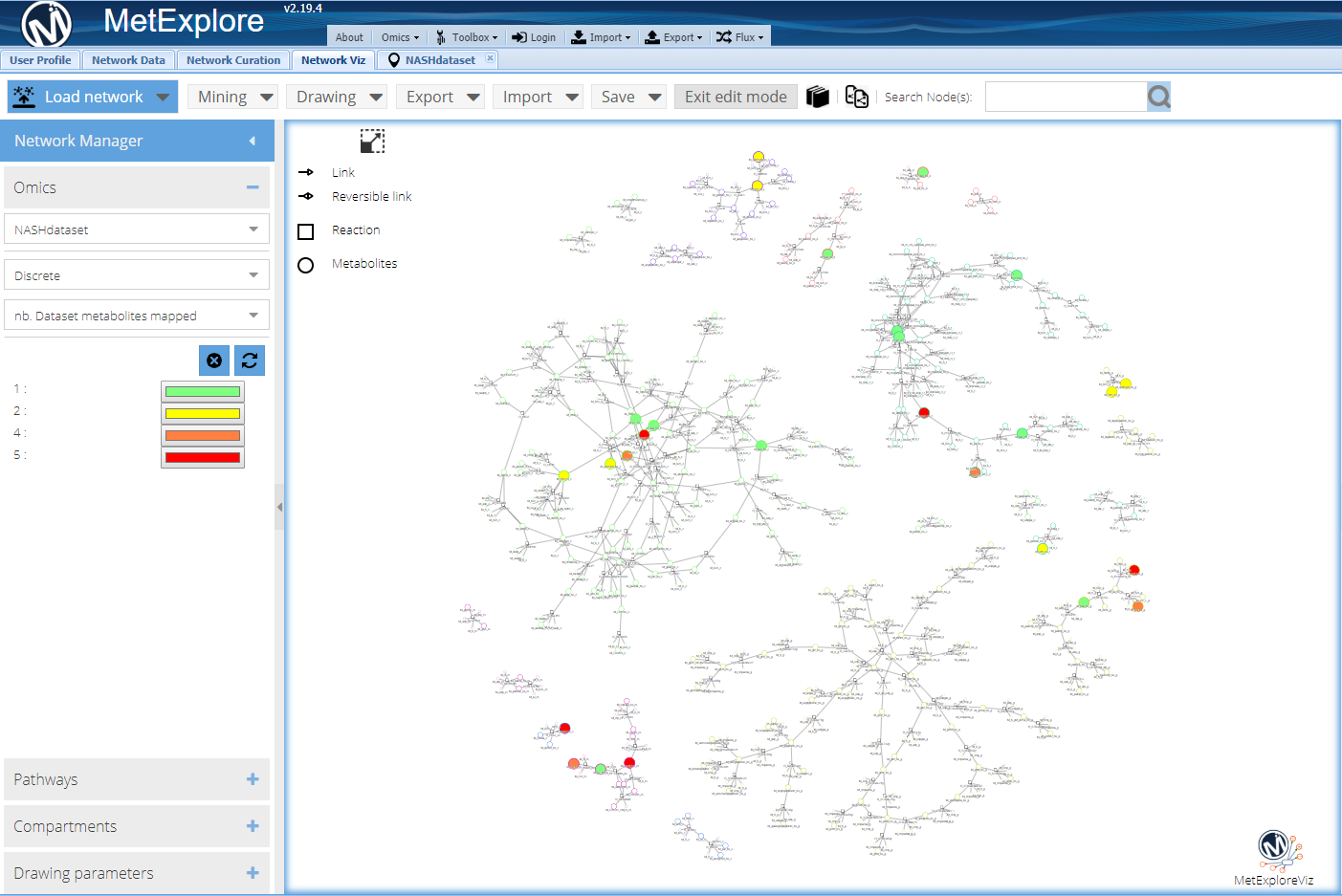


1. *Subnetwork visualization in MetExplore with highlighted mapped metabolites*

You can additionally highlight cellular compartments or metabolic pathways.

You can observe that some part of the subnetwork (for instance the sphingolipid metabolism pathway) are less covered by the NASH metabolites than others.


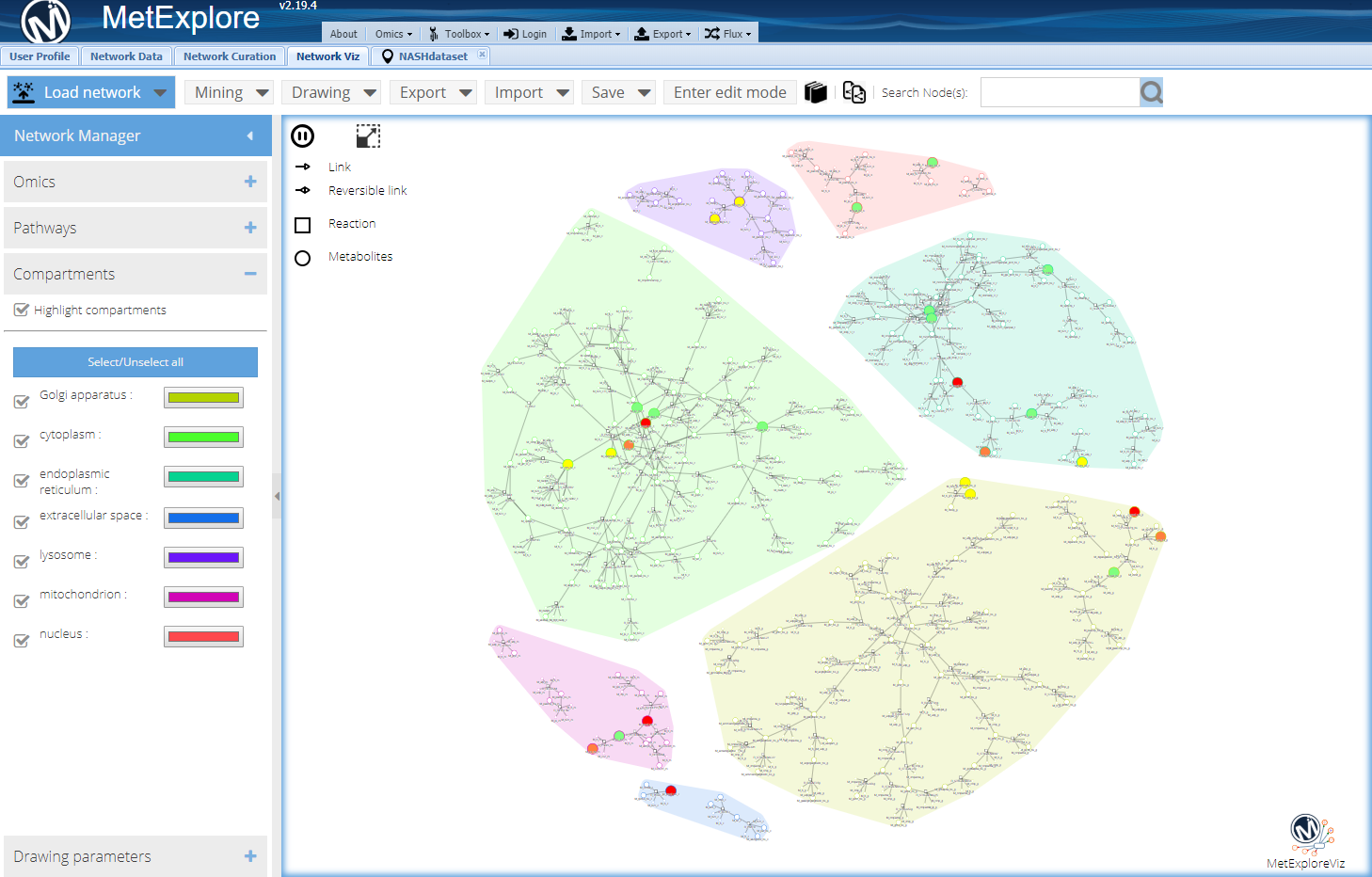

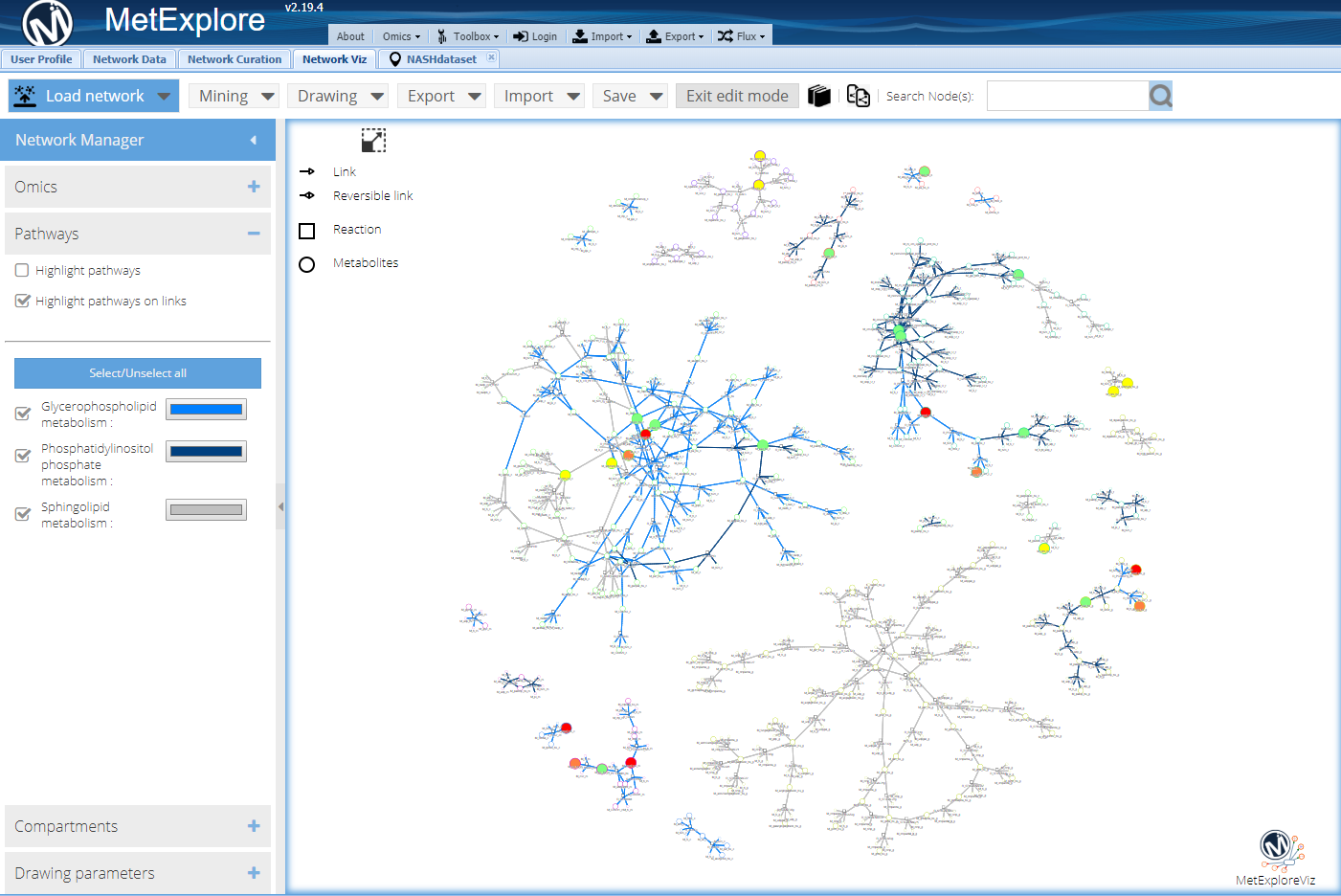


1. *Subnetwork visualization in MetExplore with highlighted mapped metabolites
   and highlighted compartments and pathways*

Visualizations can be exported as image (PNG, JPEG or SVG) using the "Export" menu in the "Network Viz" panel. They can also be saved as JSON file to be imported back and edit in MetExplore in further sessions: for that, use the "Save" menu.

Note that the mapping is not exported with the visualization and should be exported separately using the "Save Mapping in File" button in the mapping window (see section 2.3).

# References

1. Chiappini F, Coilly A, Kadar H, Gual P, Tran A, Desterke C, et al. Metabolism dysregulation induces a specific lipid signature of nonalcoholic steatohepatitis in patients. Sci Rep. 2017;7: 46658. doi:10.1038/srep46658

2. Swainston N, Smallbone K, Hefzi H, Dobson PD, Brewer J, Hanscho M, et al. Recon 2.2: from reconstruction to model of human metabolism. Metabolomics. 2016;12: 109. doi:10.1007/s11306-016-1051-4

3. Caspi R, Billington R, Ferrer L, Foerster H, Fulcher CA, Keseler IM, et al. The MetaCyc database of metabolic pathways and enzymes and the BioCyc collection of pathway/genome databases. Nucleic Acids Res. Oxford University Press; 2016;44: D471–D480. doi:10.1093/nar/gkv1164

4. BioCyc. https://biocyc.org/ [Internet].

5. KEGG. https://www.genome.jp/kegg/pathway.html [Internet].

6. Juty N, Ali R, Glont M, Keating S, Rodriguez N, Swat M, et al. BioModels: Content, Features, Functionality, and Use. CPT Pharmacometrics Syst Pharmacol. John Wiley & Sons, Ltd; 2015;4: 55–68. doi:10.1002/psp4.3

7. BioModels. https://www.ebi.ac.uk/biomodels/ [Internet].

8. Hucka M, Finney A, Sauro HM, Bolouri H, Doyle JC, Kitano H, et al. The systems biology markup language (SBML): a medium for representation and exchange of biochemical network models. Bioinformatics. 2003;19: 524–31.

9. Hucka M, Smith LP. SBML Level 3 package: Groups, Version 1 Release 1. J Integr Bioinform. 2016;13: 290. doi:10.2390/biecoll-jib-2016-290

10. Thiele I, Swainston N, Fleming RMT, Hoppe A, Sahoo S, Aurich MK, et al. A community-driven global reconstruction of human metabolism. Nat Biotechnol. 2013/03/05. 2013;31: 419–425. doi:nbt.2488 [pii]10.1038/nbt.2488
